# Strategy, Progress, and Challenges of Drug Repurposing for Efficient Antiviral Discovery

**DOI:** 10.3389/fphar.2021.660710

**Published:** 2021-05-04

**Authors:** Xinlei Li, Tao Peng

**Affiliations:** State Key Laboratory of Respiratory Disease, Sino-French Hoffmann Institute, College of Basic Medicine, Guangzhou Medical University, Guangzhou, China

**Keywords:** drug repurposing, emerging virus, antivirals, broad spectrum, COVID-19

## Abstract

Emerging or re-emerging viruses are still major threats to public health. Prophylactic vaccines represent the most effective way to prevent virus infection; however, antivirals are more promising for those viruses against which vaccines are not effective enough or contemporarily unavailable. Because of the slow pace of novel antiviral discovery, the high disuse rates, and the substantial cost, repurposing of the well-characterized therapeutics, either approved or under investigation, is becoming an attractive strategy to identify the new directions to treat virus infections. In this review, we described recent progress in identifying broad-spectrum antivirals through drug repurposing. We defined the two major categories of the repurposed antivirals, direct-acting repurposed antivirals (DARA) and host-targeting repurposed antivirals (HTRA). Under each category, we summarized repurposed antivirals with potential broad-spectrum activity against a variety of viruses and discussed the possible mechanisms of action. Finally, we proposed the potential investigative directions of drug repurposing.

## Introduction

Since December 2019, a novel coronavirus disease 2019 (COVID-19) has rapidly spread all over the globe to cause a pandemic (Li et al., 2020b; Zhu et al., 2020). The pneumonia causative agent was identified to be a new coronavirus, severe acute respiratory syndrome coronavirus 2 (SARS-CoV-2). As of April 6, 2021, more than 130 million cases have been confirmed globally, including approximately 2.85 million deaths. The still ongoing pandemic represents the most recent example of how emerging or re-emerging human or zoonotic viruses pose a threat to public health. These viruses include but not limited to Ebola virus (EBOV), Zika virus (ZIKV), West Nile virus (WNV), yellow fever virus (YFV), dengue virus (DENV), henipaviruses (Nipah, Hendra), SARS-CoV, Middle East respiratory syndrome (MERS-CoV), Lassa virus (LASV), Crimean-Congo hemorrhagic fever virus (CCHFV), Rift Valley fever virus (RVFV), chikungunya virus (CHIKV), human immunodeficiency virus (HIV) and influenza A virus (IAV). We listed six viral families in which a number of viruses have merged or remerged in recent years to have caused or potentially cause an epidemic or pandemic, including *Coronaviridae*, *Filoviridae*, *Flaviviridae*, *Arenaviridae*, *Nairoviridae*, and *Orthomyxoviridae*. The genome structure, important viruses, and key features regarding virus-host interactions are summarized in Table 1.

**TABLE 1:** Important emerging or remerging viruses.

Virus family	Genome	Important viruses	Key features/Virus-host interactions	Ref
*Coronaviridae*	ss (+) RNA; 26–32 kb	SARS-CoV, SARS-CoV-2, MERS-CoV, HCoV-229 E, HCoV-OC43, HCoV-NL63, HCoV-HKU1	Enveloped viruses; case fatality rate: 30% (MERS-CoV), 10% (SARS-CoV), 3% (SARS-CoV-2); receptor: ACE2 (SARS-CoV, SARS-CoV-2); DPP4 (MERS-CoV); S protein proteolytic cleavage by cathepsins or TMPRSS2 is necessary for infection; RNA proofreading is viable due to the exoribonuclease activity	de Wit et al. (2016); Chen (2020)
*Flaviviridae*	ss (+) RNA; 9.6–12.3 kb	DENV, ZIKV, YFV, WNV	Enveloped viruses; cause hemorrhagic fever, liver damage, congenital malformations (microcephaly); transmission by vectors like mosquitos or ticks	Mukhopadhyay et al. (2005); Barrows et al. (2018)
*Filoviridae*	ss (−) RNA; 19 kb	EBOV, MARV	Enveloped filamentous virions can exceed to 14,000 nm in length; cause fatal viral hemorrhagic fevers; case fatality rate: from 25 to 90%; DC-SIGN, or integrins as attachment factor; receptor: NPC1 (EBOV)	—
*Arenaviridae*	ss (−) RNA; segmented	LASV, JUNV	Enveloped viruses; case fatality rate: 20–30% (JUNV), 1% (LASV); entry factors: Alpha-dystroglycan, LAMP1 (LASV); cause hemorrhagic fever; virus spreads through rodents	Jae et al. (2014); Pontremoli et al. (2019)
*Nairoviridae*	ss (−) RNA; segmented	CCHFV	Enveloped viruses with circular genome; case fatality rate: 10–40% (CCHFV); virus entry is clathrin-, pH- and cholesterol dependent; cause hemorrhagic fever; transmission by vectors like ticks	Simon et al. (2009); Zivcec et al. (2016)
*Orthomyxoviridae*	ss (−) RNA; segmented	IAV (H1N1, H2N2, H5N1, H3N2, H7N9, … )	Enveloped viruses; genome reassortment is common; case fatality rate varies, 2–3% (1918 H1N1)	Ramos and Fernandez-Sesma (2012)

The emerging or remerging virus outbreak has emphasized the urgent need for preventative or treatment regimens. Vaccines are recognized as a preferred promising line of defense. However, vaccine development is a complex process and multiple challenges are involved in light of the fact that the pathogens that need to be confronted may display high genetic variability (e.g., HIV) or an identity hardly predicted in advance (e.g., SARS-CoV-2 or ZIKV). Thus, unprecedented demands have emerged on antivirals that can be rapidly available in clinical practices. In the absence of a vaccine available to use, hepatitis C virus (HCV) is supposed to be eliminated in the use of the direct-acting antivirals, which probably represents the first virus to be cured by antivirals. That strengthens the promising potential of antivirals in terms of virus treatment.

Drug repurposing (also called drug repositioning) is a strategy for identifying new uses for approved or investigational drugs that beyond the original indicative scope to facilitate antiviral development. Typically, antiviral discovery development is time and resource-consuming, which involves three major stages including drug discovery (3–6 years), preclinical studies in experimental animal models (about 3 years), clinical trials in humans from phase I to III (about 5 years). Finally, if a therapeutic succeeds to pass all the processes, it needs to get approved by the appropriate agency. It is estimated that only 5% of the candidate molecules are finally approved and up to 3 billion dollars are consumed. Given that the repurposed drugs have been proven to be safe in humans, drug repurposing likely can skip phase I and probably the phase II clinical trials. Thus, the attrition rate to be a novel antiviral is reduced, although the phase III trial is still needed. Remdesivir, an adenosine analog to inhibit EBOV RNA-dependent RNA polymerase (RdRp) (Tchesnokov et al., 2019), is the latest example. Although remdesvir did not show therapeutic activity against EBOV infection in a real-world phase III clinical trial (Nakkazi, 2018), remdesivir shows potent antiviral activity against SARS-CoV-2, SARS-CoV, and MERS-CoV *in vitro* or *in vivo* in preclinical animal models (de Wit et al., 2020; Wang et al., 2020a). Two randomized phase III clinical trials indicate that patients who received remdesivir had a shorter time to recover (Spinner et al., 2020; Wang et al., 2020c), based upon which the U.S. Food and Drug Administration (FDA) has approved remdesivir for use in COVID-19 patients, less than 1 year after the outbreak of the pandemic. From the above example, drug repurposing could significantly facilitate antiviral development for emergency use. Given the urgent need for therapeutics for emerging or re-emerging viruses and a great number of approved or developmental therapeutics, drug repurposing represents a better way for antiviral discovery. In this review, we discussed the strategies of drug repurposing for antiviral development, summarized the promising drug candidates that have the antiviral potency with broad-spectrum activity, and analyzed the possible caveats of this strategy of drug discovery.

## Strategies to Develop Repurposed Antivirals

**FIGURE 1:**
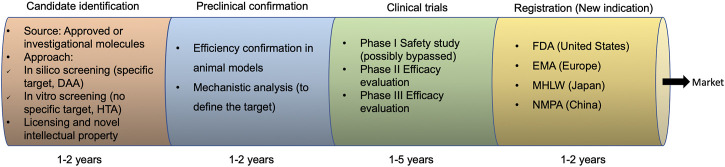
Drug repurposing development process. DAA, direct-acting antivirals; HTA, host-targeting antivirals; FDA, Food and Drug Administration; EMA, the European Medicines Agency; MHLW, Ministry of Health, Labor, and Welfare; NMPA, National Medical Products Administration.

A typical drug repurposing strategy comprises four steps (Figure 1), including the identification of a candidate therapeutic for the new indication as an antiviral; antiviral efficiency confirmation and/or mechanistic analysis in preclinical animal models; antiviral efficacy evaluation in clinical trials (phase I may be not prerequisite if sufficient safety data has already been obtained as parts of the original indication); and approval of the novel indication by government agencies such as the FDA, the European Medicines Agency (EMA), Ministry of Health, Labor and Welfare (MHLW) of Japan, and National Medical Products Administration (NMPA) of China.

## Approaches for Antiviral Repurposing

The identification of the right drug for the new indication is crucial. The major approaches involve high throughput *in silico* or *in vitro* screening. The *in silico* screening is commonly used for the identification of a compound that binds to the given target, commonly a virally encoded protein, such as RNA-dependent RNA polymerase (Patel and Kukol, 2017). The *in vitro* screening involves the high throughput antiviral screening, leading to the subsequent validation for the most potent candidates. These candidates can target host proteins or viral proteins (Kouznetsova et al., 2014; Chopra et al., 2016; Xu et al., 2016; Li et al., 2017c). For either approach, compound libraries, in particular those with approved molecules, are needed (Table 2). These include the Drugbank library, NIH Clinical Compound (NCC) Collection (van Cleef et al., 2013), the Prestwick Chemical Library (Ulferts et al., 2016), the Library of Pharmacologically Active Compounds (LOPAC) (Hu et al., 2014), a library of approved drugs that were assembled by the NIH Chemical Genomics Centre (NCGC) called the NCGC Pharmaceutical Collection (NPC) (Huang et al., 2011), and the ReFRAME (Repurposing, Focused Rescue, and Accelerated Medchem) Library (Janes et al., 2018). Recently, the LOPAC and ReFRAME drug libraries were successfully used for the discovery of the SARS-CoV-2 antiviral candidates (Riva et al., 2020).

**TABLE 2:** Compound library for drug repurposing.

Library	Library scale	Introduction	Refs
Prestwick chemical library	1,520	99% approved drugs (FDA, EMA and other agencies)	Ulferts et al. (2016)
NCATS pharmaceutical collection (or NCGC pharmaceutical Collection)	∼3,500	2,500 approved molecules, plus about 1,000 investigational compounds	Huang et al. (2011)
ReFRAME compound library	∼12,000	Containing nearly all small molecules that have reached clinical development or undergone significant preclinical profiling, 38% of which are approved drugs	Janes et al. (2018); Riva et al. (2020)
Library of pharmacologically active compounds (LOPAC), sigma	1,280	Biologically annotated collection of inhibitors, receptor ligands, pharma-developed tools, and approved drugs	Hu et al. (2014)
NIH clinical collection	727	All have a history of use in human clinical trials and known safety profiles	van Cleef et al. (2013)

## Categories of Repurposed Antivirals

**FIGURE 2:**
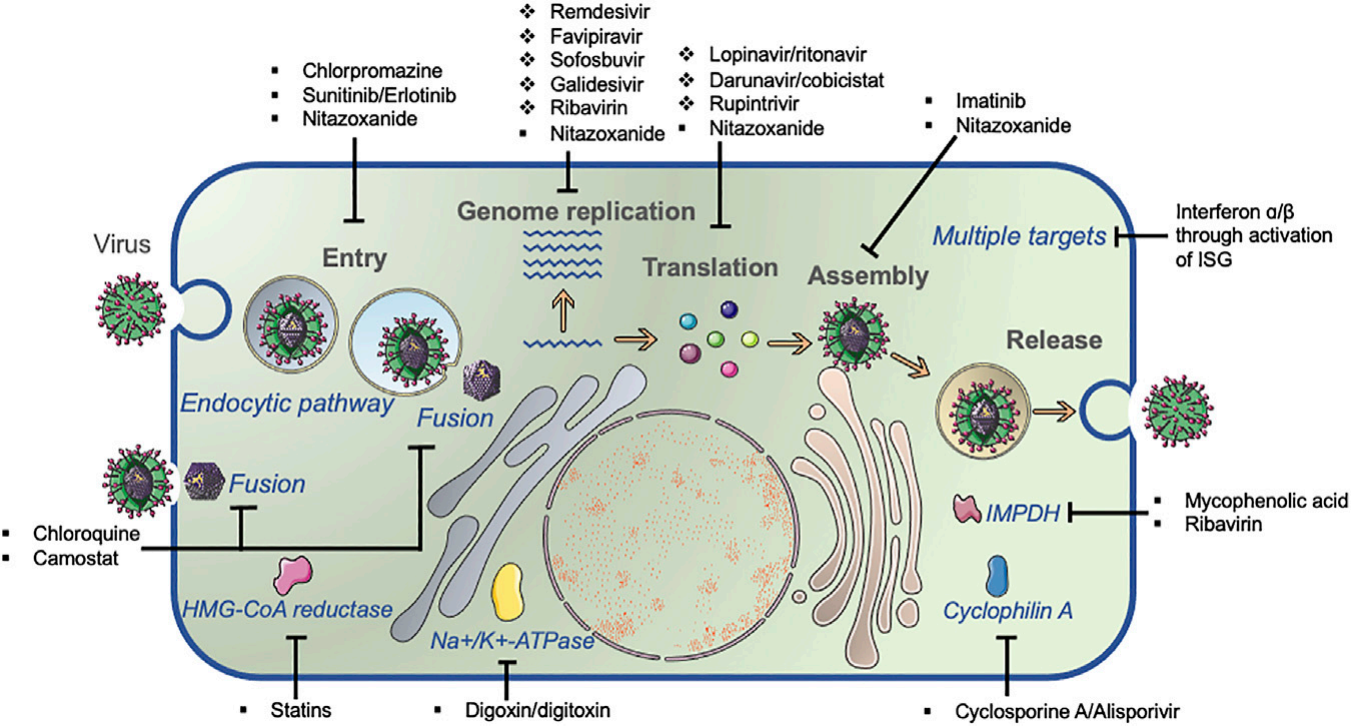
Common viral lifecycle and broad-spectrum antiviral identification. The common viral lifecycle comprises three steps: viral entry, genome replication, and virus assembly/release. Direct-acting antivirals (DAA, ❖) and host-targeting antivirals (HTA, ■) inhibit virus replication by targeting viral protein and host molecules that are required for virus replication, respectively.

Based on the origin and feature of the repurposed antiviral targets, two major categories are divided: direct-acting repurposed antiviral (DARA) and host-targeting repurposed antiviral (HTRA) repurposing. The representative antivirals with repurposed potentials are summarized in Figure 2.

### Direct-Acting Repurposed Antiviral (DARA)

A large majority of antivirals approved by the FDA are direct-acting antivirals (DAA) other than host-targeting agents (HTA) (Chaudhuri et al., 2018). DARAs contain antiviral activity relying on structural similarity or identical enzymatic activity of virally encoded targets, particularly viral polymerase, protease, reverse transcriptase, or viral proteins with ion channel activity. Below we reported the advances in repurposed antivirals targeting the two important viral enzymes, RdRp and protease.

#### RdRp Inhibitors

##### Remdesivir (GS-5734)

Remdesivir was an investigational compound in the class of nucleotide analogs, which was originally developed to treat *Filoviridae* members EBOV or Marburg infection and rapidly pushed through clinical trials due to the EBOV epidemic in West Africa from 2013 through 2016. However, in August 2019, remdesivir was announced to be less effective than the other two monoclonal antibody regimens (Mulangu et al., 2019). It has also been found to show antiviral activity against other RNA viruses such as *Pneumoviridae* member RSV (EC50 = 0.019 μM); *Paramyxoviridae* Nipah virus (EC50 = 0.029 μM), Hendra virus (EC50 = 0.055 μM), parainfluenza type three virus (EC50 = 0.018 μM), MV (EC50 = 0.037 μM) and MuV viruses (EC50 = 0.79 μM); *Arenaviridae* JUNV (EC50 = 0.47 μM), LASV (EC50 = 1.48 μM); some *Flaviviridae* viruses like Kyasanur Forest disease virus (KFDV) (EC50 = 1.8 μM), Omsk Hemorrhagic Fever virus (OHFV) (EC50 = 1.2 μM), Tick-borne encephalitis (TBEV) (EC50 = 2.1 μM), and *Coronaviridae* including MERS-CoV (EC50 = 0.074 μM), SARS-CoV (EC50 = 0.069 μM), and SARS-CoV-2 (EC50 = 0.77 μM) (Warren et al., 2016; Lo et al., 2017; Sheahan et al., 2017a; Choy et al., 2020) (Table 3). The parent nucleoside of remdesivir, GS-441524 (1′-cyano substituted adenine nucleoside analog or Nuc) also shows a broad-spectrum but less effective antiviral activity against infections of coronaviruses like MERS-CoV and feline coronavirus (Warren et al., 2016; Pedersen et al., 2019).

**TABLE 3:** Approved or investigational direct-acting antivirals with repurposed potential against other virus infections.

Category	Agent name	Primary indication	Broad antiviral activity				Clinical trials	Ref
			Virus name	EC50/EC90 (μM)	CC50 (μM)	SI		
Viral RdRp inhibitor	Remdesivir	Antiviral (EBOV, no approval)	EBOV	0.07/0.22 (Huh7 cells)	3.7	52.86	Phase III failed	Warren et al. (2016)
			JUNV	0.47/2.8	N.D.	N.D.		Warren et al. (2016)
			MERS-CoV	0.074/N.D.	>10	>135		Sheahan et al. (2017b)
			SARS-CoV	0.069/N.D.	>10	>144		Sheahan et al. (2017b)
			SARS-CoV-2	0.77/1.76	>100	>129.87	Approved for hospitalized COVID-19 patients	Wang et al. (2020b)
			RSV	0.021/0.059	6.195	395		Lo et al. (2017)
			NiV	0.029/0.053	8.294	286		Lo et al. (2017)
	Ribavirin	Antiviral (HCV, RSV)	HCV	8.4/N.D.	108	12.86	Approved	Ortega-Prieto et al. (2013)
			RSV	69.5/N.D.	N.D.	N.D.	Approved	Kim et al. (2017)
			HBV	N.D./N.D.	N.D.	N.D.	Phase I NCT04356677; phase II NCT04276688; phase III NCT04392427	Isorce et al. (2016)
			HEV	6.9/50.38	N.D.	N.D.		Todt et al. (2018)
			ZIKA	23/281	N.D.	N.D.		Kamiyama et al. (2017)
			LASV	2.47/N.D.	>50	>20		Welch et al. (2016)
			EBOV	5.34/N.D.	>50	>9		Welch et al. (2016)
			SARS-CoV	81.9/N.D.	>819	>10		Saijo et al. (2005)
			MERS-CoV	66.9/86.6	N.D.	N.D.		Falzarano et al. (2013a)
			SARS-CoV-2	109.5/N.D.	>400	3.65	Phase II/III NCT04460443, NCT04497649; phase III NCT04392427, …	Wang et al. (2020b)
	Favipiravir	Antiviral (IAV)	IAV(H1N1)	1.97/3.75	>128	>64	Approved	Sleeman et al. (2010)
			LASV	29.3/43.2	>1000	>34		Oestereich et al. (2016)
			JUNV	0.79/5.0	188	239		Furuta et al. (2013)
			CCHFV	6.37/10.18	>100	>15.7		Oestereich et al. (2014b)
			RVFV	5.0/32	>980	>196		Furuta et al. (2013)
			Rabies	32.4/N.D.	N.D.	N.D.		Yamada et al. (2016)
			RSV	N.D./36	>1600	N.D.		Jochmans et al. (2016)
			EBOV	67/110	>1000	>14.9		Oestereich et al. (2014a)
			SARS-CoV-2	61.88/N.D.	>400	>6.46	Phase III: NCT04425460, NCT04373733; phase IV NCT04359615.	Wang et al. (2020b)
			WNV	53/N.D.	N.D.	N.D.		Morrey et al. (2008)
			YFV	180/330	>6370	>19		Julander et al. (2009)
			ZIKA	22/N.D.	>637	>26		Zmurko et al. (2016)
			EV-71	68.74/N.D.	>1000	>14.55		Wang et al. (2016b)
	Sofosbuvir	Antiviral (HCV)	HCV	0.032–0.13/N.D.	N.D.	N.D.	Approved	Han et al. (2019)
			YFV	4.2/N.D.	381	90		de Freitas et al. (2019)
			DENV	1.4/6.4	>100	>71		Xu et al. (2017)
			CHIKV	1/N.D.	402	402		Ferreira et al. (2019)
			ZIKA	1.37/12.3	>200	>145		Bullard-Feibelman et al. (2017)
			HEV	1.97/N.D.	>100	>51		Netzler et al. (2019)
			HBV	—	—	—	Phase II NCT03312023	—
			SARS-CoV-2	—	—	—	Phase II/III: NCT04460443, NCT04443725; phase IV NCT04498936; …	—
	Galidesivir	Antiviral (EBOV, investigational)	EBOV	11.8/25.4	>11,800	>100	Preclinical	Warren et al. (2014)
			MARV	4.4/10.5	1065	242	Phase I NCT03800173	Warren et al. (2014)
			SUDV	3.4/10.3	>3400	>100		Warren et al. (2014)
			TBEV	0.95/N.D.	N.D.	N.D.		Eyer et al. (2019)
			YFV	14.1/46.8	>14,100	>100	Phase I NCT03891420	Warren et al. (2014)
			WNV	2.33/N.D.	>100	>42		Eyer et al. (2017)
			DENV	32.8/89.3	>9710	>296		Warren et al. (2014)
			ZIKA	3.8/18.2	N.D.	N.D.		Julander et al. (2017)
			RVFV	41.6/98.0	>41,600	>100		Warren et al. (2014)
			LASV	43.0/>100	>4300	>100		Warren et al. (2014)
			RSV	11.0/25.7	>980	>89		Warren et al. (2014)
			IAV(H1N1)	10.7/17	>3167	>296		Warren et al. (2014)
			SARS-CoV	57.7/>95	>17,080	>296		Warren et al. (2014)
			SARS-CoV-2	—	—	—	Phase I NCT03891420	—
Viral protease inhibitor	Lopinavir/ritonavir	Antiviral (HIV)	HIV (lopinavir)	0.018/N.D.	N.D.	N.D.	Approved	Masse et al. (2007)
			HIV (ritonavir)	0.046/N.D.	N.D.	N.D		Masse et al. (2007)
			SARS-CoV (lopinavir)	17.1/N.D.	>32	>2		de Wilde et al. (2014)
			MERS-CoV (lopinavir)	8.0/N.D.	24.4	3.1		de Wilde et al. (2014)
			SARS-CoV-2 (lopinavir)	26.63/N.D.	49.75	1.87	Phase III: NCT04372628, NCT04321174; phase IV: NCT04350684, NCT0435067; …	Choy et al. (2020)
	Rupintrivir	Antiviral (HRV, investigational)	HRV-100	0.022/0.032	N.D.	N.D.	Phase II completed	Binford et al. (2005)
			Echovirus-6	0.051/0.094	N.D.	N.D.		Binford et al. (2005)
			CVB2	0.022/0.088	N.D.	N.D.		Binford et al. (2005)
			CVA16/860 F	0.015/N.D.	>50	>3500		Zhang et al. (2013)
			EV71/695 F	0.014/N.D.	>50	>3500		Zhang et al. (2013)
			HCoV-229e	0.3/N.D.	>500	>1500		Kim et al. (2012)
			TGEV	2.5/N.D.	>500	>200		Kim et al. (2012)
			BOC	15.3/N.D.	>500	>32		Kim et al. (2012)
			Norovirus/Norwalk	0.32/1.5	>50	>150		Rocha-Pereira et al. (2014)

Remdesivir shows prophylactic or therapeutic potency when administrated in SARS-CoV-infected mice, in which reduced viral load in lung and improved clinical symptoms and respiratory function was observed (Sheahan et al., 2017a). A similar prophylactic or therapeutic potency of remdesivir against MERS-CoV was seen in macaques or mouse models (de Wit et al., 2020; Sheahan et al., 2020). Remdesivir efficiently inhibits SARS-CoV-2 replication *in vitro* (Wang et al., 2020b), and was used as a compassionate use in the first COVID-19 case in the United States (Holshue et al., 2020) before large-scale clinical studies (NCT04280705; NCT04292899; NCT04292730; NCT04257656) were launched. One large-scale study in which hospitalized COVID-19 patients were given remdesivir for 10 days showed significantly shortened time to recovery (Beigel et al., 2020). Another study indicated that remdesivir treatment in moderate COVID-19 patients for 5 days led to symptom improvement significantly higher than the standard care group (Spinner et al., 2020). Contrarily, a smaller scale study only found remdesivir resulted in a marginally but numerically faster time to clinical improvement (Wang et al., 2020c). Based upon these clinical studies, the full and conditional use of remdesivir in hospitalized COVID-19 patients was approved by FDA in October 2020. Although World Health Organization (WHO) recommends against it, based on the interim result of the WHO Solidarity Trial.

Mechanistically, remdesivir exerts the antiviral activity through competing with ATP that is supposed to incorporate into viral RdRp for RNA replication. It results in delayed EBOV and MERS-CoV RNA chain termination at the fifth and third position, respectively after the initiation site (Warren et al., 2016; Tchesnokov et al., 2019; Gordon et al., 2020).

##### Ribavirin (RBV)

RBV is on the WHO’s list of essential medicines, it is licensed to treat RSV infection (Committee on Infectious Diseases, 1993), or HCV infection in combination with interferon (IFN)-α or direct-acting antivirals (AASLD-IDSA HCV Guidance Panel, 2018). RBV is also effective against other hepatotropic viruses including HBV(Galban-Garcia et al., 2000) and HEV (Kamar et al., 2014; Kamar et al., 2019) in clinical studies, although no convincing activity against HBV was obtained in cell culture systems (Isorce et al., 2016). Ribavirin was clinically used to treat a variety of viral hemorrhagic fevers, including Lassa fever (McCormick et al., 1986), Crimean-Congo hemorrhagic fever (Fisher-Hoch et al., 1995), and Hantavirus infection (Ogg et al., 2013) alone or in combination with favipiravir, even though RBV might be effective only at early stages (Johnson et al., 2018; Eberhardt et al., 2019).

The clinical use of RBV as a supplement to other agents like corticosteroid for SARS-CoV treatment was documented in China and Canada (Peiris et al., 2003), while RBV had an EC50 of 81.9 μM *in vitro* (Saijo et al., 2005). RBV is also effective to inhibit MERS-CoV with an EC50 ranging from 66.9 μM (16.33 μg/ml) to 169.7 μM (41.45 μg/ml) *in vitro* (Falzarano et al., 2013a). RBV alongside IFN-α was reported to reduce the hospital mortality rate from 70 to 29% in hospitalized MERS patients at 14 days after admission (Omrani et al., 2014). RBV also shows antiviral activity against SARS-CoV-2 *in vitro* with an EC50 of 109.5 μM (Wang et al., 2020b), while another report did not find the favorable effect of RBV (Choy et al., 2020). The higher EC50 of RBV against either MERS-CoV or SARS-CoV-2 may be due to the reduced RBV uptake (Ibarra and Pfeiffer, 2009) or inabilities to accumulate sufficient amounts of phosphorylated RBV metabolites required for the effective RBV antiviral actions (Shah et al., 2010). As of early January 2021, at least seven clinical trials (phase I NCT04335123; phase II NCT04494399; phase II NCT04563208; phase II NCT04605588; NCT04664010; phase II NCT04276688; phase II/III NCT04402203) have been launched to investigate the efficacy of RBV alone or in combination with other agents for COVID-19 treatment.

Mechanistically, at least two types of antiviral machinery may be involved. Upon the uptake into cells, RBV is metabolized to form a purine RNA nucleotide-like form, which interferes with viral RNA polymerases, leading to hypermutation of RNA that reduces the viability and is lethal to RNA viruses. RBV is also an inhibitor of inosine-5′-monophosphate dehydrogenase (IMPDH), which is essential for the *de novo* synthesis of guanosine-5′-monophosphate (GMP). RBV structure may interfere with the RNA capping process that relies on natural guanosine to prevent RNA degradation. Upon the inhibition of IMPDH, RBV can lower the intracellular pool of GTP, and DNA virus replication is then inhibited. Alternatively, RBV has the potential to reduce cell death, affect type 1 cytokine production or inflammatory response, which may help combat HBV, HCV, or LASV infection (Tam et al., 1999; Oestereich et al., 2016).

##### Favipiravir

Favipiravir (6-fluoro-3-hydroxy-2-pyrazinecarboxamide) was firstly designed to inhibit influenza RdRp, despite of different serotypes and strains of influenza A, B, or C (Furuta et al., 2013). Favipiravir has been approved since 2014 in Japan for emergent use to treat influenza. In addition, favipiravir has shown a broad antiviral activity against other negative sense RNA viruses such as RSV (*Pneumoviridae*, EC90 = 36 μM), CCHFV (*Nairoviridae*, EC50 = 6.37 μM), LSAV (*Arenaviridae*, EC50 = 29.3 μM), JUNV (*Arenaviridae*, EC50 = 0.79 μM), Rabies virus (*Rhabdoviridae*, EC50 = 32.4 μM), EBOV (*Filoviridae*, EC50 = 67 μM), or positive sense RNA viruses like SARS-CoV-2 (*Coronaviridae*, EC50 = 61.88 μM), *Flaviviridae* ZIKA (EC50 = 22 μM), WNV (EC50 = 53 μM), YFV (EC50 = 180 μM), and enterovirus EV71 (*Picornaviridae*, EC50 = 68.74 μM) (Morrey et al., 2008; Oestereich et al., 2014a; Oestereich et al., 2014b; Jochmans et al., 2016; Oestereich et al., 2016; Yamada et al., 2016; Zmurko et al., 2016; Furuta et al., 2017; Wang et al., 2020b) (Table 3).

A preclinical study in EBOV-infected macaques shows a higher plasma favipiravir concentrations greater than 70–80 μg/ml or 446–509 μM were associated with reduced viral loads and extended survival rate (Guedj et al., 2018). A similar study in IFNAR−/− mice also shows a high dose [300 mg/(kg/d)] of favipiravir enhances EBOV clearance and prevents a lethal outcome (Oestereich et al., 2014a). Clinically, favipiravir showed good tolerance in EBOV patients but no strong antiviral efficacy (Sissoko et al., 2016), possibly due to the low median trough drug concentrations (46 μg/ml or 293 μM at day 2 post-treatment) (Nguyen et al., 2017). Thus, the optimal dosage and potency of favipiravir merit further investigation.

Favipiravir exhibited antiviral activity against SARS-CoV-2 *in vitro* (Wang et al., 2020b) and showed a significantly shorter viral clearance time than the control group (4 vs. 11 days) and a higher improvement in chest imaging in a non-randomized clinical study (Cai et al., 2020). Another small-sized open-label phase II/III clinical trial (NCT04434248) also found that favipiravir enabled SARS-CoV-2 viral clearance in 62.5% of patients within 4 days, as compared to 30% of patients on a standard of care; however, the viral clearance rate by day 10 after favipiravir administration was only marginally improved (Ivashchenko et al., 2020). Although favipiravir has been approved in some countries, large-scale, placebo-controlled, double-blinded clinical trials may be still needed to further evaluate the efficacy and safety of favipiravir.

The mechanism of action of inhibiting influenza RdRp by favipiravir involves the conversion to the metabolite favipiravir ribofuranosyl-5′-triphosphate (favipiravir-RTP), which further incorporates into influenza RdRp to inhibit the polymerase activity at nanomolar to micromolar concentrations (Furuta et al., 2013). The inhibition of other viral RdRp by favipiravir may involve a similar mechanism.

##### Sofosbuvir

Sofosbuvir that targets HCV RdRp NS5B from 15 subtypes in six genotypes with an EC50 ranging from 0.032 to 0.13 μM is an approved oral direct-actin antiviral to treat chronic hepatitis C (Han et al., 2019). A cocktail treatment regimen containing sofosbuvir and HCV protease NS3/4A inhibitors has already been approved for pan-genotypic HCV infection. Sofosbuvir shows antiviral effects against other virus members in *Flaviviridae* family, such as ZIKA (EC50 = 4.25 μM) (Bullard-Feibelman et al., 2017; Mumtaz et al., 2017; Sacramento et al., 2017), DENV(Sacramento et al., 2017), CHIKV (EC50 = 1 μM) (Ferreira et al., 2019), and YFV (EC50 = 4.2 μM) (de Freitas et al., 2019). Strikingly, HEV, another hepatotropic virus but evolutionally distant from HCV, was reported to be susceptible to sofosbuvir (EC50 = 1.97 μM) (Todt et al., 2018; Netzler et al., 2019) (Table 3). A phase II clinical trial of sofosbuvir for HBV treatment (phase II NCT03312023) is also under investigation. Besides, the binding residue of sofosbuvir on coronavirus RdRp is conserved among SARS-CoV, SARS-CoV-2, and MERS-CoV(Jacome et al., 2020), although sofosbuvir did not exhibit the inhibitory effect against MERS-CoV RdRp in a cell-based reporter assay (Min et al., 2020). Sofosbuvir binds to SARS-CoV-2 RdRp and inhibits virus infection in lung and brain cells (Elfiky, 2020a), and clinical trials have initiated in multiple countries (phase II NCT04561063, phase II NCT04532931, phase II/III NCT04460443; phase II/III NCT04497649; phase III NCT04530422, phase III NCT04535869, phase IV NCT04498936).

##### Galidesivir (BCX4430, Immucillin-A)

Galidesivir, an imino-C-nucleoside analog, was originally developed to combat EBOV infection (Warren et al., 2014). Galidesivir strongly inhibits EBOV RdRp activity *in vitro* and post-exposure intramuscular administration of galidesivir protects mice or macaques against Ebola virus or Marburg disease (Warren et al., 2014). Currently, a phase I clinical trial (NCT03800173) for Marburg disease is being performed.

Galidesivir was subsequently identified to exhibit broad-spectrum antiviral effectiveness against other RNA virus families like *Flaviviridae* members TBEV (EC50 = 0.95 μM), YFV (EC50 = 14.1 μM), WNV (EC50 = 2.33 μM), DENV (EC50 = 32.8 μM), and ZIKA (EC50 = 3.8 μM), *Arenaviridae* (LASV, EC50 = 43.0 μM), *Phleboviridae* (RVFV, EC50 = 41.6 μM), *Pneumoviridae* (RSV, EC50 = 11.0 μM), *Orthomyxoviridae* (IAV H1N1, EC50 = 10.7 μM), and *Coronaviridae* MERS-CoV (EC50 = 68.4 μM) and SARS-CoV (EC50 = 57.7 μM) (Warren et al., 2014; Eyer et al., 2017; Julander et al., 2017; Westover et al., 2018; Eyer et al., 2019). Preclinical studies showed intramuscular or intraperitoneal administration of galidesivir in Syrian golden hamsters effectively limited systemic RVFV infection and improved survival outcomes (Westover et al., 2018). Galidesivir also showed anti-ZIKA activity in a lethal mouse model even when the treatment was initiated during the peak of viremia (Julander et al., 2017). Despite the anti-coronavirus activity *in vitro*, and the predicted strong binding of galidesivir with SARS-CoV-2 RdRp (Elfiky, 2020b), an early stage clinical showed that treatment with galidesivir offered COVID-19 patients no benefit compared to a placebo.

#### Viral Protease Inhibitor

##### Lopinavir/ritonavir (LPV/r)

LPV/r is a fixed-dose combination for HIV prevention and treatment. It combines LPV with a low dose of ritonavir (RTV), both of which are HIV protease inhibitors. In HIV-1 NL4-3 infection system *in vitro*, LPV and RTV have an EC50 of 0.018 and 0.046 μM, respectively, against HIV-1, but LPV has a much higher potency than RTV does (EC50 = 0.015 μM for LPV vs. 0.349 μM for RTV) for HIV-2 (Masse et al., 2007). RTV is also a very potent inhibitor of intestinal and hepatic cytochrome P450 3A4 (Eagling et al., 1997), which is involved in LPV catabolism. As with LPV/r, darunavir/cobicistat (DRV/c) is also a dose-fixed combination containing HIV protease inhibitor DRV and CYP3A enzymatic antagonist cobicistat (Masse et al., 2007). Low doses of RTV or cobicistat could slow down the breakdown of HIV protease inhibitors, thereby greatly increases its blood concentration.

Some viruses, like SARS-CoV, MERS-CoV, and SARS-CoV-2, encode proteases, which are structurally and functionally similar to HIV protease. LPV shows an EC50 of 17.1, 8.0, and 26.63 μM, respectively against the three coronaviruses (de Wilde et al., 2014; Choy et al., 2020) (Table 3). LPV/r combination shows greater anti-MERS-CoV activity than LPV does (EC50 of 8.5 vs. 11.6 μM) *in vitro* (Sheahan et al., 2020). LPV/r alongside IFN-β shows improved clinical and pathological features in a nonhuman primate MERS model (Chan et al., 2015), while prophylactic or therapeutic LPV/r-IFN-β treatment only slightly improves the disease outcomes in patients (Sheahan et al., 2020). Similarly, SARS patients receiving LPV/r were found to have an improvement in respiratory syndrome (Chu et al., 2004). However, the potency of LPV/r for SARS-CoV is only effective if administrated early but not as rescue or salvage therapy (Chan et al., 2003). Despite of its potential for COVID-19 treatment, a randomized trial showed the hospitalized COVID-19 patients did not benefit from LPV/r therapy (Cao et al., 2020).

##### Rupintrivir (AG-7088)

Rupintrivir is a peptidomimetic compound inhibiting viral protease activity, it is designed to combat human rhinovirus (HRV, belonging to family Piconaviridae) infection. Rupintrivir shows potent *in vitro* activity against all 48 HRV serotypes tested, with a range of EC50s of 0.007 to 0.104 μM (Binford et al., 2005). A phase II placebo-controlled randomized, double-blind trial experimentally shows the biosafety and potential efficacy in volunteers (Hayden et al., 2003). Because of a lack of efficacy in natural HRV infection in a subsequent clinical trial, further development of rupintrivir was suspended.

Rupintrivir has shown antiviral activity against a spectrum of viruses that encodes 3C or 3C-like protease, for instance, rupintrivir exhibits antiviral activities against multiple enteroviruses, including EV71 (strain 695F, EC50 = 0.014 μM), coxsackievirus B2 (CVB2, EC50 = 0.022 μM), CVA16 (strain 860F, EC50 = 0.015 μM) (Hung et al., 2011). Two studies show rupintrivir exhibits cross-genotypic inhibitory activity against either human or mouse norovirus, a member in the family *Caliciviridae*, with the EC50 of 0.32 and 13 μM, respectively (Kim et al., 2012; Rocha-Pereira et al., 2014). A molecular modeling study shows rupintrivir is capable to bind with SARS-CoV main proteinase 3CL^pro^ (Anand et al., 2003); however, rupintrivir fails to show good activity at even 100 μM, although some rupintrivir derivatives show better potency (IC50 = 11–39 μM) (Shie et al., 2005). Rupintrivir exerts an antiviral effect on coronaviruses including CoV-229E (EC50 = 0.3 μM), transmissible gastroenteritis virus (TGEV, EC50 = 2.5 μM), bovine coronavirus (BCV, EC50 = 15.3 μM) (Table 3). Rrupintrivir also showed inhibition for SARS-CoV-2 main protease with a 50% inhibitory concentration of 68 ± 7 µM (Vatansever et al., 2021).

Rupintrivir has poor aqueous solubility and low oral bioavailability in animals, the hydrolyzed metabolites are reportedly 400-fold less active than rupintrivir but predominates the biotransformation pathway. The above features may limit its potential clinical application.

### Host-Targeting Antiviral (HTA) Repurposing

HTA repurposing identifies antivirals targeting to host proteins, functions, or pathways, which are required for virus life cycle including viral entry, genome replication, protein translation, and virus assembly and release. As the entire viral life cycle cannot be completed without cells, HTA may exhibit broad antiviral activity against different viruses. Based on the essential steps of a viral life cycle, four major categories of host-targeting repurposed antivirals (HTRA) are classified as below.

#### HTRA Aiming Virus Entry Step

The first step of the viral life cycle is to enter permissive cells. Some enveloped viruses like HIV, and Nipah virus enter cells *via* direct membrane fusion with the plasma membrane, resulting in the release of nucleocapsid directly to the cytosol (Bossart et al., 2002; Wilen et al., 2012). Bacteriophages can inject their genomes alone into bacterial cells. Except for the aforementioned two mechanisms, most viruses depend on an endocytic pathway to be internalized into cells. The involved pathways include clathrin-mediated endocytosis, caveolar/lipid raft-mediated endocytosis, or micropinocytosis, through which viruses are internalized into the early endosome, intermediate endosome, and then late endosome or lysosome in a stepwise manner. Finally, the exposure of virions either naked or enveloped to low pH and proteolytic enzymes will trigger changes in the naked virions, or membrane fusion between the organelle and enveloped viruses, to help deliver the viral genome or the intact nucleocapsid into cytosol. Aftermath, most RNA viruses replicate in different locations within the cytosol, whereas DNA viruses continue the journey to the nucleus.

##### Chlorpromazine (CPZ) and Other Dopamine Antagonists

CPZ is a phenothiazine used to treat psychotic disorders including schizophrenia or manic-depression in adults. CPZ can treat in children severe behavioral problems like attention deficit hyperactivity disorder. CPZ is also indicated to treat anxiety before surgery, nausea and vomiting, and chronic hiccups that do not improve following other treatments (Lopez-Munoz et al., 2005). CPZ is on the list of WHO’s essential medicines, among the most effective and safest medicines. CPZ antagonizes dopamine receptors, which are divided into two classes based on which G-protein they are coupled: the D1-like class (including D1 and D5) and the D2-like class, which comprises D2, D3, and D4 receptors. CPZ can bind to and block two types of dopamine receptors, in particular D2 dopamine receptors, exerting antipsychotic activity.

CPZ has proved to inhibit clathrin-mediate endocytosis by preventing the assembly of the clathrin-coated pit on the cell surface (Wang et al., 1993). Thus, CPZ and other dopamine receptor antagonists show antiviral activity against a broad spectrum of viruses that use clathrin-mediated endocytosis to enter cells. These viruses include HIV(Bosch et al., 2008), rubella virus (Kee et al., 2004), human adenovirus (HAdV) (Diaconu et al., 2010), EV71 (Hussain et al., 2011), HAV (Rivera-Serrano et al., 2019), HEV (Yin et al., 2016a), HCV (Blanchard et al., 2006), DENV (Carro et al., 2018), ZIKA (Persaud et al., 2018; Li et al., 2020a), CSFV (Shi et al., 2016), CCHFV (Simon et al., 2009; Ferraris et al., 2015b), SFV (Pohjala et al., 2011), EBOV (Bhattacharyya et al., 2010), MERS-CoV (de Wilde et al., 2014), and SARS-CoV (Inoue et al., 2007).

HIV can either enter cells through direct viral membrane fusion with the plasma membrane, or cell-to-cell transmission and viral synapses between T cells. The latter type of HIV entry is sensitive to CPZ treatment, suggesting the involvement of clathrin-mediated endocytosis (Bosch et al., 2008). Viruses within *Flaviviridae* family, such as HCV, DENV, ZIKA, and CSFV also enter cells dependent on clathrin-mediated endocytosis and are susceptible to CPZ treatment (Zhu et al., 2012; Shi et al., 2016). Cell surface FcγR was reported to be required for antibody-dependent enhancement of DENV or ZIKV infection (Khandia et al., 2018). Interestingly, the viral entry mediated by FcγRII needs the formation of clathrin-coated vesicles whilst FcγRI-dependent viral entry is independent of clathrin (Carro et al., 2018). On the contrary, naked and enveloped viruses may comparably be sensitive to CPZ. HAV and HEV had been recognized as naked viruses until recently the membrane-trapped viral particles were identified (Feng et al., 2013; Yin et al., 2016a). The naked and enveloped HAV or HEV are both sensitive to CPZ treatment (Yin et al., 2016a; Rivera-Serrano et al., 2019), suggesting the clathrin-mediated endocytosis is equally needed. Coronaviruses such as MERS-CoV and SARS-CoV share the same clathrin-mediated endocytosis for virus entry. In light of this, clinical studies have initiated (phase II/III NCT04354805; phase III NCT04366739) to evaluate the safety and effectiveness of CPZ for COVID-19 treatment, although observative clinical studies have suggested that CPZ at the prescribed dose may not be clinically effective for COVID-19 (Hoertel et al., 2021).

##### Sunitinib, Erlotinib (Receptor Tyrosine Kinase Inhibitors)

Sunitinib and erlotinib are inhibitors to receptor tyrosine kinases (RTK) that play important roles in both tumor angiogenesis and tumor cell proliferation. Sunitinib has been approved for the treatment of cancers, such as gastrointestinal stromal cell tumor, renal cell carcinoma, and imatinib-resistant gastrointestinal stromal tumor; while erlotinib is licensed to treat non-small cell lung cancer, and pancreatic cancer (Hartmann and Kanz, 2008; Neveu et al., 2015). Erlotinib is on the list of WHO’s essential medicines.

The major antiviral mechanism of sunitinib involves the inhibition of adaptor protein 2 (AP2)-associated protein kinase 1 (AAK1), which phosphorylates membrane trafficking adaptor proteins AP-1 and AP-2 to enhance the binding with clathrin-associated cargos for bidirectional transport and endocytosis from the plasma membrane, respectively (Ricotta et al., 2002). The inhibition of AAK1 thereby inhibits virus entry, or assembly and release. For instance, sunitinib reportedly inhibits DENV entry and infectious virus release but not RNA replication (Bekerman et al., 2017). In a multiple cycle infection system, the EC50 against DENV1 is 0.6 μM, similar EC50s (0.3–1.2 μM) of sunitinib against other members in the family *Flaviviridae* (HCV, ZIKV, other DENV serotypes) were reported (Bekerman et al., 2017) (Table 4). Sunitinib is also effective against infections of other viruses including EBOV (EC50 = 0.47 μM), CHIKV (EC50 = 4.67 μM), JUNV (EC50 = 4.8 μM), HIV (EC50 = 0.8 μM), and RSV (EC50 < 0.12 μM) (Bekerman et al., 2017). Albeit sunitinib and erlotinib combinations showed no efficacy in murine models of DENV and EBOV infection (Bekerman et al., 2017).

**TABLE 4:** Approved and investigational host-targeting antivirals with repurposed potential against virus infection.

Agent name	Primary indication/mechanism of action	Repurposed antiviral activity						Clinical trials	Ref
		Category	Virus name	EC50/EC90 (μM)	CC50 (μM)	SI	Mechanism of action		
Chlorpromazine (CPZ)	Anti-psychotic; CPZ antagonizes dopamine receptors to exert antipsychotic activity	Virus entry inhibitor	SARS-CoV	8.8/N.D.	24.3	2.8	CPZ and other dopamine receptor antagonize clathrin-mediated endocytosis which is required for some virus entry	Phase II/III NCT04354805; phase III NCT04366739	de Wilde et al. (2014)
			MERS-CoV	4.9/N.D.	21.3	4.3			de Wilde et al. (2014)
			SARS-CoV-2	—	—	—			-
			HCV	N.D./N.D.	N.D.	N.D.			Blanchard et al. (2006)
			DENV	N.D./N.D.	N.D.	N.D.			Carro et al. (2018)
			ZIKA	N.D./N.D.	N.D.	N.D.			Khandia et al. (2018)
			CSFV	N.D./N.D.	N.D.	N.D.			Shi et al. (2016)
			CCHFV	10.6/N.D.	30	2.8			Ferraris et al. (2015b)
			SFV	15.7/N.D.	67.3	4.5			Pohjala et al. (2011)
			EBOV	N.D./N.D.	N.D.	N.D.			Bhattacharyya et al. (2010); Du et al. (2020)
			HAV	N.D./N.D.	N.D.	N.D.			Rivera-Serrano et al. (2019)
			HEV	N.D./N.D.	N.D.	N.D.			Yin et al. (2016a)
Chloroquine (CQ)	Anti-malaria; lysosomotropic CQ accumulates in acidic digestive vacuole inside red blood cells, where CQ binds to hemes to form a toxic product resulting in cell lysis and ultimately parasite cell autodigestion		HCV	<50/N.D.	>100	>2	As a lysosomotropic agent, CQ blocks the membrane fusion between virus and lysosomes to inhibit virus entry	Phase III: NCT04447534, NCT04360759; phase IV: NCT04362332, NCT04331600; …	Ashfaq et al. (2011)
			DENV	N.D./N.D.	N.D.	N.D.			Farias et al. (2014)
			ZIKA	7.25/N.D.	>30	>4			Ianevski et al. (2018)
			CCHFV	39.4/N.D.	1000	26.9			Ferraris et al. (2015a)
			SARS-CoV	6.5/N.D.	>100	>15			Dyall et al. (2014)
			MERS-CoV	6.28/N.D.	>10	>1.5			Dyall et al. (2014)
			SARS-CoV-2	1.13/N.D.	>100	>88.5			Wang et al. (2020b)
			EBOV	1.57/3.35	N.D.	N.D.			Du et al. (2020)
			eHAV	N.D./N.D.	N.D.	N.D.			Feng et al. (2013)
			HIV	<0.9/N.D.	N.D.	N.D.			Boelaert et al. (1999)
Sunitinib/erlotinib	Anti-tumor; inhibiting tumor cell growth by inhibiting receptor tyrosine kinase (RTK) pathways		HCV(sunitinib)	1.2/N.D.	>10	>8.3	Sunitinib/erlotinib inhibits RTKs including AAK1 or EGFR, which is invovled in intracellular vesicle trafficking that is used by some virus to enter cells	Phase I/II NCT02380625	Bekerman et al. (2017)
			HCV(erlotinib)	0.6/N.D.	>15	>25			Bekerman et al. (2017)
			DENV1(sunitinib)	0.6/N.D.	>10	>16.7			Bekerman et al. (2017)
			DENV1(erlotinib)	1.9/N.D.	>20	>10.5			Bekerman et al. (2017)
			ZIKV(sunitinib)	0.51/N.D.	14.1	27.6			Bekerman et al. (2017)
			ZIKV(erlotinib)	6.28/N.D.	>30	>4.7			Bekerman et al. (2017)
			EBOV(sunitinib)	0.47/N.D.	>10	>21.2			Bekerman et al. (2017)
			EBOV(erlotinib)	2.88/N.D.	15	5.2		Phase I/II NCT02380625	Bekerman et al. (2017)
			CHIKV(sunitinib)	4.67/N.D.	11.9	2.5			Bekerman et al. (2017)
			CHIKV(erlotinib)	0.7/N.D.	30	42.9			Bekerman et al. (2017)
			JUNV(sunitinib)	4.8/N.D.	10.4	2.2			Bekerman et al. (2017)
			JUNV(erlotinib)	1.7/N.D.	>20	>11			Bekerman et al. (2017)
			HIV(sunitinib)	0.8/N.D.	>20	>15			Bekerman et al. (2017)
			HIV(erlotinib)	2/N.D.	>20	>10			Bekerman et al. (2017)
			RSV(sunitinib)	<0.12/N.D.	12.5	>100			Bekerman et al. (2017)
			RSV(erlotinib)	<0.12/N.D.	>30	>250			Bekerman et al. (2017)
			HBV(erlotinib)	N.D./N.D.	N.D.	N.D.			Gan et al. (2020)
			SARS-CoV-2	—	—	—		Phase I: NCT02134886, NCT00890747; phase I/II: NCT02380625, NCT01835938; phase II NCT00521092; …	—
Camostat	Chronic pancreatitis and postoperative reflux esophagitis; inhibiting transmembrane protease, serine 2 (TMPRSS2)		SARS-CoV	∼1/∼5	>500	>500	Camostat is an inhibitor to TMPRSS2 which is required for entry of coronaviruses or influenza	Phase II/III NCT04455815; phase III NCT04355052; phase IV NCT04338906; …	Hoffmann et al. (2020b)
			MERS-CoV	∼1.5/∼5	>500	>300			Hoffmann et al. (2020b)
			SARS-CoV-2	∼1/∼5	>500	>500			Hoffmann et al. (2020b)
			IAV-A	4.4/N.D.	>1000	>200			Hosoya et al. (1992)
			IAV-B	11.7/N.D.	>1000	>85			Hosoya et al. (1992)
Statins	Hypercholesterolemia; statins are HMG-CoA reductase inhibitors to reduce cholesterol biosynthesis	Virus replication inhibitor	DENV	11.9/N.D.	53.6	4.5	Statin inhibits replicase formation of flaviviruses, reverse transcription of HIV; impairs EBOV glycoprotein processing	Phase II NCT02841774; phase III NCT03037372	Martinez-Gutierrez et al. (2011)
			ZIKA (cerivastatin)	0.02/N.D.	0.37	18.1			Españo et al. (2019)
			ZIKA (lovastatin)	14.59/N.D.	38.76	2.66			Españo et al. (2019)
			HCV (lovastatin)	N.D./N.D.	N.D.	N.D.			Ye et al. (2003)
			HIV	N.D./N.D.	N.D.	N.D.			Giguère and Tremblay (2004); Amet et al. (2008)
			HBV (simvastatin)	5.2/N.D.	>30	>5.7			Okuyama-Dobashi et al. (2015)
			EBOV (fluvastatin)	0.89/2.48	N.D.	N.D.			Du et al. (2020)
			RSV	N.D./N.D.	N.D.	N.D.			Gower and Graham (2001); Ravi et al. (2013a)
			CVB3	N.D./N.D.	N.D.	N.D.			Werner et al. (2014)
			MV	N.D./N.D.	N.D.	N.D.			Robinzon et al. (2009)
			SARS-CoV-2	—	—	—		Phase III: NCT04486508, NCT04472611; phase IV NCT02735707; …	—
Digoxin	Heart failure; the cardiac glycoside can block the Na^+^/K^+^ ATPase ion pump activity to raise the intracellular Ca^2+^ level to improve the cardiac failure		HSV	0.13/N.D.	10.21	78.54	Digoxin impedes the immediate-early or early gene expressions of herpes viruses; impaires JEV or EBOV RNA replication or virus entry; may also inhibit coronavirus entry; inhibits arenaviruses whose replication depends on Na^+^/K^+^ ATPase activity	—	Su et al. (2008)
			HSV (digitoxin)	0.05/N.D.	10.66	213		—	Su et al. (2008)
			HCMV	0.036/N.D.	0.45	12.6		—	Kapoor et al. (2012)
			HAdV	N.D./N.D.	N.D.	N.D.		—	Grosso et al. (2017)
			HIV	0.045/0.1	>0.1	>2		—	Wong et al. (2013)
			HBV (digitoxin)	0.093/N.D.	1.7	18.9		—	Okuyama-Dobashi et al. (2015)
			JEV	0.10/N.D.	>100	>969.9		—	Guo et al. (2020)
			CHIKV	0.0488/N.D.	>10	>200		—	Ashbrook et al. (2016)
			SINV	0.1989/N.D.	N.D.	N.D.		—	Ashbrook et al. (2016)
			RRV	0.1265/N.D.	N.D.	N.D.		—	Ashbrook et al. (2016)
			RSV	0.026/N.D.	0.839	32.3		—	Norris et al. (2018)
			LCMV (ouabain)	0.0058/N.D.	0.0289	4.99		—	Iwasaki et al. (2018)
			LASV	N.D./N.D.	N.D.	N.D.		—	Iwasaki et al. (2018)
			JUNV	N.D./N.D.	N.D.	N.D.		—	(Iwasaki et al., 2018)
			Reovirus	0.1339/N.D.	N.D.	N.D.		—	Ashbrook et al. (2016)
			VSV	0.2387/N.D.	N.D.	N.D.		—	Ashbrook et al. (2016)
			EBOV	0.32/2.44	N.D.	N.D.		—	Du et al. (2020)
			MERS-CoV (ouabain)	N.D./N.D.	N.D.	N.D.		—	Burkard et al. (2015)
			SARS-CoV-2	0.043/N.D.	>10	>232	—	—	Cho et al. (2020)
Mycophenolic acid (MPA)	Immuno-suppression; MPA inhibits IMPDH to cause the intracellular guanosine depletion, resulting in immunosuppressive activity in lymphocyte		CHIKV	1.5/N.D.	>200	>130	MPA exerts antiviral activity through IMPDH-mediated depletion of intracellular guanosine, on which viral genome replication replies	Phase II NCT03262441	Pohjala et al. (2011)
			HCV	0.68/N.D.	73.8	108.5			Ye et al. (2012)
			DENV	0.3/N.D.	N.D.	N.D.			Diamond et al. (2002)
			WNV (New York isolate)	2.8/0.94	>312	>111			Morrey et al. (2002)
			ZIKV	0.1–1/N.D.	N.D.	N.D.			Barrows et al. (2016)
			HBV	N.D./N.D.	N.D.	N.D.			Gong et al. (1999); Ying et al. (2000)
			HIV	N.D./N.D.	N.D.	N.D.			Chapuis et al. (2000)
			HEV	N.D./N.D.	N.D.	N.D.			Wang et al. (2014)
			VacV	0.7/N.D.	48	68.6			Smee et al. (2001)
			HuNoV	N.D./N.D.	N.D.	N.D.			Dang et al. (2017)
			Rotavirus	0.885/N.D.	438.07	495.05			Yin et al. (2016b)
			IAV (H1N1)	1.51/N.D.	>50	>33			To et al. (2016)
			MERS-CoV	0.53/8.15	>32	>195.12			Chan et al. (2013)
			SARS-CoV-2	0.15/N.D.	—	—			Han et al. (2021)
Cyclosporine a (CsA)	Immuno-suppression; CsA is an inhibitor to peptidylprolyl isomerase cyclophilin a (CyPA), causing the block of T cell activation though the cypa-calcineurin-nf-at pathway		HCV	0.33/N.D.	N.D.	N.D.	CyPA-dependent: CyPA interacts with flavivirus proteins, IAV M protein, nucleocapsid and Nsp1 proteins of SARS-CoV. CyPA-independent: CsA inhibits HBV entry, IAV replication	Phase III completed	Koizumi et al. (2017)
			DENV	N.D./N.D.	N.D.	N.D.		NCT04451239; phase I NCT04412785; phase II NCT04492891; phase IV NCT0439253	Qing et al. (2009)
			MERS-CoV	3.6/N.D.	26.4	7.3			de Wilde et al. (2017)
			SARS-CoV	1.3/N.D.	>50	>38.5			de Wilde et al. (2017)
			SARS-CoV-2	0.46/3.1	>10	>21			Softic et al. (2020)
			HIV	N.D./N.D.	N.D.	N.D.			Franke et al. (1994); Hatziioannou et al. (2005)
			HBV	1.12/N.D.	>10	>8.9			Watashi et al. (2014)
			IAV	1.45/N.D.	>20	>13			Hamamoto et al. (2013)
Imatinib	Anti-tumor; imatinib inhibits tyrosine kinase c-Abl to block tumor cell proliferation	Virus assembly/release inhibitor	EBOV	N.D./N.D.	N.D.	N.D.	Imatinib decreases the budding or release of EBOV, VacV, and DENV; imatinib also inhibits CVB entry	Phase II: NCT04357613, NCT04346147; phase III: NCT04394416, NCT04422678; …	Garcia et al. (2012)
			DENV	N.D./N.D.	N.D.	N.D.			Clark et al. (2016)
			MERS-CoV	17.7/N.D.	>50	>2.8			Dyall et al. (2014)
			SARS-CoV	9.82/N.D.	>50	>5			Dyall et al. (2014)
			SARS-CoV-2	4.86/N.D.	37.3	7.7			Han et al. (2021)
			VacV	N.D./N.D.	N.D.	N.D.			Reeves et al. (2011)
Interferon α/β	Approved for antiviral (HCV, HBV) and multiple sclerosis treatment; IFN induces the production of inteferon-stimulated genes through JAK-STAT pathway	Inhibition of multiple targets	HCV (IFN-α)	*2.5/10	>10,000	>4000	IFN exerts the broad-specrum antiviral activity by inducing ISG production, which may inhibit each step of the viral life cycle		Okuse et al. (2005)
			HBV(IFN-α2)	^#^7754/N.D.	N.D.	N.D.		Phase I NCT03294798; phase II NCT03575208; phase IV NCT03357822, NCT04035837; …	Chen et al. (2020)
			HIV	N.D./N.D.	N.D.	N.D.		Phase 1/2 NCT0247143	Iyer et al. (2017)
			EBOV (IFN-β)	*74/N.D.	N.D.	N.D.			McCarthy et al. (2016)
			EBOV (IFN-α)	*748/Ν.D	N.D.	N.D.			McCarthy et al. (2016)
			DENV	*182.1/N.D.	N.D.	N.D.			Pires de Mello et al. (2018a)
			ZIKV	*407.8/N.D.	N.D.	N.D.			Pires de Mello et al. (2018b)
			SARS-CoV(IFN-α)	*4950/N.D.	>10,000	>2			Cinatl et al. (2003)
			SARS-CoV(IFN-β)	*95/N.D.	>10,000	>105			Cinatl et al. (2003)
			MERS-CoV(IFN-α-2b)	*58.08/320.11	N.D.	N.D.			Falzarano et al. (2013a)
			SARS-CoV-2 (IFN-α or β)	*1.35 or 0.76/N.D.	N.D.	N.D.		Phase III: NCT04492475, NCT04320238; phase IV: NCT04350671, NCT04254874; …	Mantlo et al. (2020)
Nitazoxanide	Anti-parasite; nitazoxanide interferences with the pyruvate:ferredoxin oxidoreductase (PFOR) enzyme-dependent electron transfer reaction to disrupt parasite enegery metabolism		IAV (H1N1/PR8)	3.25/N.D.	>163	>50	Nitazoxanide inhibits morphogenesis of IAV and rotavirus; post-entry steps of SeV and RSV; HBV DNA replication; viral protease activity of ZIKV or coronavirus; induce innate immune genes	Phase II NCT03905655	Rossignol et al. (2009)
			SeV	1.63/N.D.	>163	>100			Rossignol (2014)
			RSV	0.98/N.D.	>163	>166			Rossignol (2014)
			HCV	0.68/3.03	123.7	181			Korba et al. (2008)
			JEV	0.39/N.D.	60	154			Shi et al. (2014)
			ZIKV	1.48/4.0	77	52			Li et al. (2017c)
			HIV	1.63/N.D.	>100	>50			Tan et al. (2012)
			HBV	0.39/2.7	>325	>833			Korba et al. (2008)
			Rotavirus	6.5/N.D.	>163	>25			La Frazia et al. (2013)
			CHIKV	2.96/N.D.	25	8.45			Wang et al. (2016c)
			MHV	1/N.D.	>10	>10			Cao et al. (2015)
			SARS-CoV-2	2.12/N.D.	35.53	16.76		Phase III: NCT04382846, NCT04392427; phase IV: NCT04498936, NCT04406246; …	Wang et al. (2020b)

Note: N.D. not determined. # pg/ml; * IU/ml for IFN unit.

EGFR is involved in multiple virus entry processes such as DNA viruses HBV, HPV, and RNA viruses HCV, RSV, and porcine reproductive and respiratory syndrome virus in cell cultures (Lupberger et al., 2011; Wang et al., 2016a; Iwamoto et al., 2019; Lingemann et al., 2019; Mikuličić et al., 2019). Specifically, EGFR mediates HCV entry by regulating CD81–claudin-1 associations and viral glycoprotein-dependent membrane fusion (Lupberger et al., 2011). EGFR reportedly associates with sodium taurocholate cotransporting polypeptide (NTCP), the HBV receptor on the hepatocyte cell surface, and inhibition of EGFR dramatically impairs HBV virion internalization (Iwamoto et al., 2019; Gan et al., 2020). However, a recent clinical study suggests that HBV reactivation may occur in lung cancer patients receiving erlotinib treatment (Yao et al., 2019). Therefore, the safety and efficacy of sunitinib/erlotinib need to be cautiously investigated.

##### Chloroquine (CQ) (Lysosomotropic Agents)

CQ is a medication primarily used to treat or prevent a non-resistant malaria infection, it is also occasionally used for amebiasis treatment. Additionally, CQ has shown anti-inflammatory properties for the clinical management of some autoimmune diseases such as rheumatoid arthritis and lupus erythematosus (Rainsford et al., 2015). CQ is on the list of WHO’s essential medicines. The anti-malarial mechanism of action involves the lysosomotropic feature, which allows CQ to accumulate in an acidic digestive vacuole inside red blood cells, where CQ binds to hemes to form a toxic product resulting in cell lysis and ultimately parasite cell autodigestion. Also, because of the involvement of lysosomes in the autophagy process, the inhibition by CQ of lysosomal enzymes leads to the accumulation of the autophagy cargos that are supposed to break down in lysosomes, leading to the impairment of autophagy machinery.

Due to the lysosomotropic feature, CQ or other agents like hydroxychloroquine (HCQ) and Bafilomycin A, accumulate in lysosomes, acidic endosomes, or trans-Golgi network vesicles to elevate the pH and subsequently inhibit the residential hydrolase activity (Thome et al., 2013). Such an inhibition in lysosomal function leads to the uptake impairment of a panel of viruses. These include HCV (Ashfaq et al., 2011), DENV (Farias et al., 2014; Farias et al., 2015), ZIKA (Li et al., 2017b; Shiryaev et al., 2017), CCHFV (Ferraris et al., 2015a), CHIKV (Khan et al., 2010), MERS-CoV (Dyall et al., 2014), SARS-CoV (Keyaerts et al., 2004; Vincent et al., 2005), and SARS-CoV-2 (Wang et al., 2020b).

CQ exerts antiviral activity against DENV infection either *in vitro* (Farias et al., 2014) and *in vivo* in an infected monkey model (Farias et al., 2015). However, a double-blind and placebo-controlled trial in 307 dengue patients found that CQ does not reduce the duration of viremia and the viral NS1 antigenemia (Tricou et al., 2010). Although CQ shows antiviral activity against EBOV *in vitro*; however, CQ fails to protect against EBOV infection and disease pathogenesis in the *in vivo* guinea pig model (Dowall et al., 2015). CQ shows antiviral activity against some strains of HIV *in vitro* (Tsai et al., 1990) with an EC50 of 0.4–0.9 μM when treated in combination with hydroxyurea plus didanosine in a lymphocytic cell line (Boelaert et al., 1999). And HCQ exhibits anti-HIV activity *in vivo* (Sperber et al., 1995). The mechanism may involve the inhibition of cell-to-cell transmission of HIV, which requires clathrin-mediated endocytosis (Bosch et al., 2008), or the inhibition of p120 production at a post-transcriptional level, possibly by impairing hydrolases or other enzymes in acidic vesicles (Savarino et al., 2001).

CQ shows broad and good antiviral activity against coronavirus infection *in vitro*. However, no efficacy is observed to reduce SARS-CoV virus titers in a nonlethal mouse model (Barnard et al., 2006a). Although CQ and HCQ had drawn a lot of attention for the treatment of COVID-19, the safety or severe side effects of CQ or HCQ at a high dose is concerned (Borba et al., 2020; Cortegiani et al., 2020), and recent clinical studies (including WHO Solidarity trial) indicate that CQ or HCQ has little efficacy for COVID-19 (Boulware et al., 2020).

##### Camostat or Nafamostat Mesylate (TMPRSS2 Inhibitor)

Camostat and nafamostat are serine protease inhibitors that inhibit the transmembrane protease, serine 2 (TMPRSS2). Camostat is approved in Japan for the treatment of postoperative reflux esophagitis and chronic pancreatitis, while nafamostat is approved for pancreatitis, an anticoagulant in patients with disseminative blood vessel coagulation, hemorrhagic lesions, and hemorrhagic tendencies (Jia et al., 2005; Maruyama et al., 2011).

Camostat and nafamostat exhibit effectiveness against a broad spectrum of viruses from different families including IAV (Hosoya et al., 1992; Hosoya et al., 1993; Lee et al., 1996), myxoviruses (Hosoya et al., 1992), DENV (Rathore et al., 2019), MERS-CoV (Shirato et al., 2013), SARS-CoV (Kawase et al., 2012), and SARS-CoV-2 (Hoffmann et al., 2020b) (Table 4). Particularly, camostat and nafamostat are effective to inhibit type A or B IAV replication *in vitro* at a micromolar level, while showing no efficacy against other respiratory viruses tested including RSV and parainfluenza virus (Hosoya et al., 1992). Additionally, camostat mesylate at an ED50 (the survival rate of influenza virus-infected chick embryos by 50%) of 0.8 μg/g can increase the survival rate of influenza virus-infected chick embryos (Lee et al., 1996). The mechanism of camostat antiviral activity involves the inhibition of hemagglutinin (HA) cleavage, which is essential for the process of IAV infection and is achieved intracellularly or extracellularly by host proteases like TMPRSS2 (Yamaya et al., 2015).

SARS-CoV and SARS-CoV-2 both use angiotensin-converting enzyme 2 (ACE2) as a receptor, whereas MERS-CoV utilizes DDP4 as the receptor for entry (Li et al., 2003; Raj et al., 2013; Lan et al., 2020). These three coronaviruses all use two mechanisms to enter cells. One involves the direct membrane fusion at the cell surface after the virions binding to receptors, the membrane fusion is triggered by TMPRSS2 serine protease to generate a cleaved form of spike (S) protein for fusion step (Kawase et al., 2012; Shirato et al., 2013; Yamamoto et al., 2016; Hoffmann et al., 2020a). Alternatively, these viruses enter cells through the endocytic pathway in the absence of TMPRSS2, involving the cleavage and priming of S protein by cysteine protease cathepsin B/L in acidic endosomes (Simmons et al., 2005; Qian et al., 2013; Hoffmann et al., 2020a). Inhibition of both TMPRSS2 serine protease and cathepsin B/L cysteine protease activities are required for full blockade of the above coronavirus infection (Kawase et al., 2012; Hoffmann et al., 2020a), while TMPRSS2 inhibition by camostat or nafamostat only results in partial impairment in virus entry (Kawase et al., 2012; Shirato et al., 2013). Strikingly, camostat at a dose of 30 mg/g shows antiviral efficacy by improving the survival rate in a lethal SARS-CoV-infected mouse model; however, a cysteine protease inhibitor SMDC256160 at a higher dose of 50 mg/g is no effective (Zhou et al., 2015). The result suggests that TMPRSS2 rather than cathepsin B/L facilitates the spread of SARS-CoV in the infected mice. It would be of interest to evaluate the treatment efficacy of camostat or nafamostat for SARS-CoV-2 infection, and multiple clinical studies have been available for camostat (phase I/II NCT04321096; phase I/II NCT04435015; phase II NCT04353284; phase II NCT04374019; phase II NCT04470544; phase II/III NCT04455815; phase III NCT04355052; phase IV NCT04338906) and nafamostat (phase II/III NCT04352400; phase II/III NCT04418128; phase II/III NCT04473053).

#### HTRA Targeting Virus Replication Step

After the viral genome is uncoated from nucleocapsid, viral genome replication and protein translation occur. Positive-sense RNA viruses, for instance, coronaviruses and flaviviruses, directly use the viral genome as a template for viral protein translation using host machinery. Negative-sense RNA viruses like filoviruses and myxoviruses, need to generate positive-sense RNA by the virally encoded polymerase, and then protein translation is initiated. Retrovirus and HBV replication involve one additional step, copying RNA to DNA by using virally encoded reverse transcriptase. DNA viruses need to use a host RNA polymerase to generate RNA from the viral DNA genome for protein translation. A number of viruses replicate in specific compartments, so-called replication organelles, in the cytoplasm, involving the aberrant lipid-rich membrane rearrangement (de Wilde et al., 2018). Specifically, some flaviviruses or alphaviruses replicate on an architecture composed of single-membrane spherules (den Boon and Ahlquist, 2010); while coronaviruses or picornaviruses form double-membrane vesicles as replicase sites (de Wilde et al., 2013; van der Schaar et al., 2016). To facilitate viral genome amplification, a variety of host proteins or related pathways are required to generate a favorable environment for virus production. These host proteins or pathways that interact with viral proteins are ideal host-targeting antivirals with a potential comprehensive antiviral efficacy.

##### Statins (HMG-CoA Reductase Inhibitors)

Statins are reversible inhibitors of 3-hydroxy-3-methylglutaryl-CoA (HMG-CoA) reductase, a rate-limiting enzyme involved in cholesterol biosynthesis. The statins are clinically approved to reduce cholesterol levels to prevent primary and secondary cardiovascular diseases. There are various forms of statins, which include lovastatin, atorvastatin, fluvastatin, pitavastatin, pravastatin, rosuvastatin, and simvastatin. Simvastatin is on the list of the WHO’s essential medicines. Statins have been reported to inhibit a panel of disparate viruses including the viruses within the family *Flaviviridae* (HCV, DENV, and ZIKV) (Ye et al., 2003; Soto-Acosta et al., 2017; Españo et al., 2019), HIV (Amet et al., 2008), HBV (Okuyama-Dobashi et al., 2015), MV (Robinzon et al., 2009), EBOV (Shrivastava-Ranjan et al., 2018), RSV (Gower and Graham, 2001), EBV (Katano et al., 2004; Cohen, 2005), PRV (Desplanques et al., 2010), SFTSV (Urata et al., 2018), and parainfluenza (Bajimaya et al., 2017), because cholesterol biosynthesis is required for the replication of these viruses.

Statins efficiently inhibit flaviviral replication either in cell cultures or in animal models. Lovastatin impairs HCV RNA replication by blocking geranylgeranylation of a host protein required for HCV replication. Statins inhibit infectious ZIKV production as well as virus spread, possibly through the inhibition at either the early stage and the post-entry steps (Españo et al., 2019). Lovastatin impairs DENV replicase complex formation or virus assembly (Martinez-Gutierrez et al., 2011; Soto-Acosta et al., 2017). In addition, lovastatin, either prophylactically or even 48 hr post-infection significantly prolongs the survival rate of DENV-infected mice (Martinez-Gutierrez et al., 2014). Lovastatin is able to inhibit HIV reverse transcriptase activity (Mazière et al., 1994), HIV attachment (Giguère and Tremblay, 2004), or HIV virion release (Amet et al., 2008). However, drug-drug interactions are reported to exist for statins and viral protease inhibitors like HCV NS3/4A antagonists or HIV protease inhibitors (Busti et al., 2004; Chauvin et al., 2013). Thus, the efficacy of statins against Flavivirus or HIV infection in the real world needs to be further assessed.

Negative strand RNA viruses are susceptible to statins. Lovastatin shows antiviral potency in RSV-infected mice prophylactically and prevents the illness-associated weight loss (Gower and Graham, 2001), which is consistent with the observation that RSV induces HMG-CoA reductase activity and lovastatin is able to inhibit RSV replication *in vitro* (Ravi et al., 2013b). Lovastatin impairs coxackie virus B3 infection through downregulating coxsackie and adenovirus receptor expression (Werner et al., 2014). Lovastatin inhibits hPIV assembly and release but no other steps (Bajimaya et al., 2017). Statins are capable of impairing EBOV glycoprotein processing and infectious EBOV production and the glycoprotein-induced attachment (Hacke et al., 2015; Shrivastava-Ranjan et al., 2018). Retrospective clinical studies found that statins may help improve the outcome in hospitalized flu patients (Rothberg et al., 2012), although no effect was observed in IAV-infected rodent models (Kumaki et al., 2012; Belser et al., 2013; Glück et al., 2013).

Statins may benefit COVID-19 patients according to a retrospective study in 13,981 COVID-19 patients (Zhang et al., 2020). Currently, at least eight clinical trials are being on the way to continue the investigation into the efficacy of statins for COVID-19 (NCT04333407; phase II NCT04348695; phase II NCT04380402; phase II/III NCT04466241; phase III NCT04486508; phase III NCT04472611; phase III NCT04343001; phase IV NCT02735707).

##### Digoxin (Ion Pump Modulator)

Digoxin is a cardiac glycoside or cardiotonic steroid, on the WHO’s list of essential medicines. Digoxin has been used to treat certain heart dysfunctions including atrial fibrillation, and other heart failures (Gheorghiade et al., 2006). Digoxin has been shown to block the Na^+^/K^+^-ATPase with an inhibitory potency around 100–200 nM (Noel et al., 2018), resulting in elevated intracellular Na^+^ level, and subsequent Ca^2+^ level *via* the sodium-calcium exchanger. Digoxin and its analogs are reported to inhibit a global type of viruses, including dsDNA virus adenovirus (Grosso et al., 2017) and HSV (Dodson et al., 2007), retrovirus HIV (Wong et al., 2018), HBV (Okuyama-Dobashi et al., 2015), positive-sense alphavirus CHIKV, SINV, and RRV (Ashbrook et al., 2016), negative-sense enveloped RNA virus VSV, dsRNA naked virus reovirus (Ashbrook et al., 2016), RSV (Norris et al., 2018), arenaviruses including LCMV, LASV, and JUNV (Iwasaki et al., 2018), filovirus EBOV (Du et al., 2020), and coronaviruses (Burkard et al., 2015) (Table 4).

Cardiac glycoside efficiently inhibits DNA virus replication. Digoxin or ouabain exhibits herpes virus, such as HSV and HCMV, at the immediate-early or early gene expression stage, the antiviral activity replies on the inhibition in Na^+^/K^+^-ATPase activity (Dodson et al., 2007; Su et al., 2008; Kapoor et al., 2012), Besides, digoxin also reportedly inhibits HSV release step (Su et al., 2008). Digoxin also inhibits HAdV DNA synthesis (Grosso et al., 2017).

Other cardiac glycosides but not digoxin are able to inhibit HBV infection *in vitro,* possibly through blocking HBV preS1 protein binding to its receptor NTCP (Okuyama-Dobashi et al., 2015). Digoxin inhibits HIV protein expression in peripheral blood mononuclear cells, by a mechanism involving the impaired activity of the CLK family of SR protein kinases (Wong et al., 2013), or the modulation of MEK1/2-ERK1/2 signaling (Wong et al., 2018). In addition, digoxin may also negatively affect HIV integration in T cells (Zhyvoloup et al., 2017). As digoxin exhibits anti-HIV activity with an excellent EC50 (1.1–1.3 nM) at which it is far below the concentration in clinical use, cardiac glycosides merit further investigation to validate the efficacy for HIV treatment.

Digoxin and ouabain at nanomolar inhibit JEV infection in multiple cell culture systems, and ouabain protects against the JEV infection-induced lethality in mice (Guo et al., 2020). The inhibitory mechanism of digoxin or ouabain against (+) ssRNA virus infection may involve impaired RNA replication or virus entry (Ashbrook et al., 2016; Guo et al., 2020).

Na^+^/K^+^-ATPase is implicated in the entry process of coronaviruses including MHV and FIPV, cardiac glycosides like ouabain or bufalin inhibit MHV, FIPV, and MERS-CoV infections, by inhibiting virus entry and membrane fusion steps (Burkard et al., 2015). By contrast, cardiac glycosides inhibit SARS-CoV-2 at the post-entry step rather than S protein-mediated virus fusion or syncytia formation (Cho et al., 2020; Musarrat et al., 2020). Despite the excellent antiviral activity of cardiac glycosides against SARS-CoV-2 *in vitro*, no clinical trials have been initiated. One of the possible concerns is the association between digoxin use and increased all-cause mortality.

##### Mycophenolic Acid (MPA) (IMPDH Inhibitor)

MPA, also called mycophenolate, is an immunosuppressant approved to prevent transplant organ rejection and to treat Crohn’s disease (van Gelder and Hesselink, 2015). MPA is a reversible, non-competitive inhibitor of IMPDH, which is the rate-limiting enzyme for the *de novo* synthesis of guanosine nucleotides that are substrates for DNA or RNA synthesis. MPA is more potent to inhibit type II than type I IMPDH enzyme, the former type expresses in activated lymphocytes, while the latter one in other most cells (Allison and Eugui, 2000). DNA or RNA virus replication replies on host guanosine pool, thus, MPA shows a broad spectrum antiviral activity against a variety of viruses, including HIV (Chapuis et al., 2000), HEV (Wang et al., 2014), HBV (Gong et al., 1999), BK polyoma virus (Acott et al., 2009), HCV and flaviviruses (DENV, WNV, JEV, and ZIKA) (Diamond et al., 2002; Morrey et al., 2002; Henry et al., 2006; Sebastian et al., 2011; Ye et al., 2012; Barrows et al., 2016; Adcock et al., 2017), orthobunyaviruses (Guama and Tacaiuma viruses) (Livonesi et al., 2007), orthopoxviruses like Vaccinia virus (VacV) and cowpox (Smee et al., 2001), rotavirus (Chan et al., 2013), SINV (Scheidel and Stollar, 1991), IAV (To et al., 2016; Cho et al., 2017; Hui et al., 2018), and MERS-CoV(Chan et al., 2013) (Table 4).

MPA displays anti-HIV activity both *in vitro* and in HIV patients (Margolis et al., 1999; Chapuis et al., 2000), and a phase II clinical study (NCT03262441) is currently under investigation. Additionally, MPA potentiates the antiviral effect of reverse transcriptase inhibitors such as abacavir (Margolis et al., 1999). The antiviral mechanisms involve the depleted guanosine pool, as well as the induction of T cell apoptosis (Chapuis et al., 2000).

MPA is also effective for HBV, at 31.2 μΜ (10 μg/ml, the therapeutic concentration in serum for immunosuppressive effect) in primary hepatocytes drastically reduces the secretion of HBV DNA and HBsAg, as well as the intracellular cccDNA level (Gong et al., 1999). Moreover, MPA and RBV, another IMPDH inhibitor, enhance the anti-HBV activity of nucleoside analogs including entecavir (Ying et al., 2000; Ying et al., 2007).

Although MPA shows anti-HCV potency *in vitro* or a mouse model (Henry et al., 2006; Pan et al., 2012; Ye et al., 2012), it fails to show antiviral efficacy in a double-blinded and placebo-controlled clinical study (Firpi et al., 2003). MPA presents anti-JEV activity *in vitro* with an EC50 of 9.68 μM (3.1 μg/ml) and up to 75% protection against the lethal challenge of JEV *in vivo* (Sebastian et al., 2011). MPA effectively dampens DENV replication with an EC50 of 0.3 μM *in vitro* (Diamond et al., 2002; Manchala et al., 2019), and similarly inhibits ZIKV with the EC50 between 0.1 and 1 μM (Barrows et al., 2016), although high cytotoxicity was also observed (Adcock et al., 2017).

MPA inhibits human and avian-originated IAV *in vitro*, including IAV-A (H1N1) (pdm09/H1/415, EC_50_ = 1.51 μM), A (H3N2), A (H5N1) (Vietnam/1194/2004, EC_50_ = 0.94 μM), A (H7N9) and IAV-B (To et al., 2016; Cho et al., 2017). MPA also shows efficacy in an H5N1-infected mouse model (Cho et al., 2017).

After a repurposed drug screening, MPA exhibited good anti-MERS-CoV activity with an EC50 (0.53 μM), EC90 (8.15 μM), and high SI value (>195.12) (Chan et al., 2013). MPA in combination with IFN-β further lowers the EC50 by 1–3 times (Chan et al., 2013). Contrarily, IMPDH inhibitors including MPA slightly enhance SARS-CoV replication in the lungs (Barnard et al., 2006b). MPA enables to inhibit SARS-CoV-2 infection in different cell cultures (Kato et al., 2020; Han et al., 2021), however, no clinical evidence is available to show the efficacy of MPA in COVID-19 patients.

##### Cyclosporine A (CsA) (Cyclophilin Inhibitor)

CsA is an immunosuppressant firstly isolated from fungus and has been approved to treat and prevent graft-versus-host disease in bone marrow transplantation, to prevent rejection of kidney, heart, and liver, or to treat autoimmune diseases like rheumatoid arthritis and psoriasis transplants (Griffiths and Voorhees, 1990; Faulds et al., 1993). CsA was recently approved as eye drops to treat dry eye disease (Mandal et al., 2019). CsA is on the WHO’s list of essential medicines. The immunomodulatory mechanism of CsA involves its binding to peptidylprolyl isomerase cyclophilin A (CyPA). The CsA-CyPA complex is able to inhibit the calcineurin phosphatase activity, the nuclear translocation of the nuclear factor of activated T cells (NF-AT), eventually block the transcription of cytokines and T cell activation (Matsuda and Koyasu, 2000). CsA and its analog like alisporivir (ALV) have shown a broad-spectrum antiviral capacity against viruses, including *Flaviviridae* members HCV and ZIKV (Watashi et al., 2003; Henry et al., 2006; Koizumi et al., 2017; Dong et al., 2019), hepatotropic viruses HBV and HEV (Wang et al., 2014; Watashi et al., 2014), HIV (Keckesova et al., 2006; Saini and Potash, 2006; Nicolás et al., 2017), coronavirus SARS-CoV, SARS-CoV-2, CoV-NL63, and MERS-CoV(de Wilde et al., 2011; Pfefferle et al., 2011; Tanaka et al., 2013; Carbajo-Lozoya et al., 2014; Guisado-Vasco et al., 2020), rotavirus (Shen et al., 2015; Yin et al., 2016b), norovirus (Dang et al., 2017), DNA virus CMV (Abdullah et al., 2018), and IAV (Hamamoto et al., 2013; Ma et al., 2016).

CsA inhibits HCV RNA replication and the antiviral capacity seems independent of its immunosuppressive effect (Watashi et al., 2003; Henry et al., 2006), as ALV, a non-immunosuppressive CsA analog, maintains the anti-HCV capacity (Crouchet et al., 2018). ALV in follow-up clinical trials (phase I to III) shows a higher sustained virological response rate than IFN and ribavirin do (Stanciu et al., 2019). However, serious side effects were more frequent in ALV-treated patients, the clinical studies were then halted. CyPA interacts with flavivirus proteins and is required for viral replication (Chatterji et al., 2009; Chatterji et al., 2010), and CsA is capable of inhibiting the infections of DENV, WNV, and YFV(Qing et al., 2009). Interestingly, CsA shows strong adulticidal activity against mosquitos, although no direct anti-ZIKV activity was found in a mosquito cell culture system (Dong et al., 2019).

CsA displays anti-HBV activity *in vitro*, and the inhibition is independent of CyPA or calcineurin (Watashi et al., 2014; Phillips et al., 2015). The inhibitory mechanism involves the block of the interactions of NTCP, the HBV entry receptor, with HBV large envelope protein (Watashi et al., 2014). CsA derivatives that maintain the anti-HBV activities but lose the impact on NTCP transporter activity have been successfully developed (Shimura et al., 2017). CyPA interacts with IAV matrix (M) protein (Liu et al., 2009), but CsA inhibits IAV or IBV infection at the steps of viral replication, IAV protein translation, or virus assembly/release, in a CyPA-independent manner (Liu et al., 2012; Hamamoto et al., 2013; Ma et al., 2016). Further preclinical and clinical studies for either virus infection are warranted later.

CyPA specifically binds to the nucleocapsid and Nsp1 proteins of SARS-CoV and is detectable in the SARS-CoV particles (Chen et al., 2005; Pfefferle et al., 2011). CsA inhibits diverse coronaviruses including SARS-CoV, MERS-CoV, HCoV-229E, HCoV-NL63, and MHV (de Wilde et al., 2011; Pfefferle et al., 2011; de Wilde et al., 2013). 16 μM CsA drastically inhibits infectious SARS-CoV production by >3log, although CsA less than 4 μM seems to have pro-viral activity (de Wilde et al., 2011). CsA inhibits SARS-CoV RNA replication or post-entry steps (Pfefferle et al., 2011; Softic et al., 2020), and the early step is also possibly affected (de Wilde et al., 2011). Despite the encouraging results in cell culture systems, treatment with RBV and ALV does not protect from SARS-CoV infection in a mouse model (de Wilde et al., 2017). 9 μM CsA treatment completely blocks the MERS-CoV-induced cytopathology in Vero cells, and CsA in combination with IFN-α display more effective anti-MERS-CoV activity (de Wilde et al., 2013; Li et al., 2018). ALV displays antiviral activity against SARS-CoV-2 with an EC50 of 0.46 μM *in vitro* (Softic et al., 2020), and CsA in a cohort study showed a 4-fold decrease in observed mortality in hospitalized COVID-19 patients (Guisado-Vasco et al., 2020). Currently, at least four clinical trials have been in the process to evaluate the efficacy of CsA or ALV to treat COVID-19 (NCT04451239; phase I NCT04412785; phase II NCT04492891; phase IV NCT04392531). More results will be available soon.

#### HTRA Targeting Virus Assembly/Release Step

After a sufficient viral structure protein pool is available, viral assembly, a dynamic process driven by programmed sequential reactions is initiated, which involves interactions between the viral genomes and viral capsid proteins, and virus-host protein associations. The newly assembled nonenveloped virions disrupt the cytoskeleton to facilitate dispersal of viral progenies, whilst enveloped viruses gain their envelope from an intracellular organelle or plasma membrane to exit the cells by a budding or exocytosis process, albeit the dividing line between nonenveloped and enveloped viruses has become blurred given that non-lytic spread mechanisms have been identified for HAV, HEV, and some enteroviruses (Feng et al., 2013; Bird et al., 2014; Chen et al., 2015; Yin et al., 2016a). The host endosomal sorting complexes required for transport (ESCRT) and autophagy machinery have emerged roles to mediate the virus release despite the envelopment.

##### Imatinib (STI-571) (c-Abl Inhibitors)

Imatinib is a 2-phenyl amino pyrimidine derivative that functions as a specific inhibitor of many tyrosine kinases, including c-Abl, c-Kit, and platelet-derived growth factor receptor. It replaces ATP in the enzymatically active site, leading to the decreased activity of these tyrosine kinases. Imatinib is a medication used to treat cancer including chronic myelogenous leukemia, acute lymphocytic leukemia, and gastrointestinal stromal tumors. Imatinib is on the list of WHO’s essential medicines. c-Abl is also implicated in the lifecycle of different viruses, and imatinib has been reported to inhibit infection of EBOV, DENV, MERS-CoV, SARS-CoV, coxsackievirus, and VacV (Table 4).

c-Abl1 inhibitor imatinib or nilotinib drastically decreases the budding or release of EBOV, as the inhibition of phosphorylation of the viral matrix protein VP40 (Garcia et al., 2012). Similarly, imatinib significantly dampens the extracellular enveloped VacV virion release without affecting cell-associated enveloped virions, and imatinib shows prophylactical or therapeutic antiviral effect in VacV-infected mice (Reeves et al., 2011). In addition, imatinib significantly inhibits DENV replication at the post-entry steps, reducing the production of infectious DENV (Clark et al., 2016).

Interestingly, imatinib appears to inhibit the entry step of group B coxsackieviruses (CVBs), blocking the aggregation of virions to the tight junction, where the virions subsequently initiate the internalization step to finally surmount the epithelial barrier (Coyne and Bergelson, 2006).

Imatinib or other c-Abl inhibitors nilotinib and dasatinib are able to inhibit MERS-CoV or SARS-CoV infection (Dyall et al., 2014). Specifically, imatinib and dasatinib show effectiveness against both viruses, while nilotinib is only effective for SARS-CoV (Dyall et al., 2014). Recently, imatinib was reported to inhibit SARS-CoV-2 in stem cell-differentiated lung organoids (EC50 = 4.86 μM) (Han et al., 2021). The detailed mechanism for this inhibition warrants further investigation. Currently, at least five clinical trials including three phase III studies (NCT04394416; NCT04422678; NCT04356495) have been carried out to investigate the treatment efficacy of imatinib for COVID-19.

##### Other Agents

Other agents that are also capable of inhibiting virus assembly/release include statins, which, as mentioned previously, inhibits virion assembly of DENV or parainfluenza, and impairs infectious HIV or EBOV release (Amet et al., 2008; Martinez-Gutierrez et al., 2011; Bajimaya et al., 2017; Shrivastava-Ranjan et al., 2018). Another example is nitazoxanide, exhibiting multiple targeting features, can inhibit assembly/release of IAV, rotavirus, and possibly paramyxoviruses (Rossignol et al., 2009; La Frazia et al., 2013; Piacentini et al., 2018).

### Repurposed Agents With Multiple Targets

Some repurposed agents have more than three potential targets, either viral or host proteins. The most documented example is IFN-α/β, which is a crucial member of innate immunity, the first line to defend pathogens including viruses. Another instance is nitazoxanide, which has shown a very broad antiviral efficiency, representing divergent antiviral mechanisms for different viruses.

#### Interferon

Almost all viruses can induce interferon response that is mediated by different sensors including cGAS for DNA viruses; RIG-I, MDA5 for RNA viruses (Tan et al., 2018). These pattern recognition receptors recognize the invaders containing pathogen-associated molecular patterns to induce IFN, which in turn secretes out of cells and binds to receptors to induce the activation of JAK-STAT pathway. As a result, a broad spectrum of interferon-stimulated genes (ISG) is induced and exert antiviral effects through different mechanisms (Liu et al., 2013; Bailey et al., 2014; Li et al., 2017a). Clearly, the ISG network is diverse and complicated, each ISG functions in concert with others, in a combinatorial or even redundant way to combat virus infection. There are three classes of IFNs, type I, type II, and type III, distinguished by their different receptors. The type I IFNs include IFN-α, IFN-β, IFN-ε, IFN-κ, and IFN-ω; type II IFN comprises IFN-γ; and type III IFNs refer to IFN-λ1 (IL-29), IFN-λ2 (IL-28A), IFN-λ3 (IL-28B), and IFN-λ4 (Stanifer et al., 2019). IFN-α has been used for clinical purposes against HCV and HBV for a long time, and IFN-β is approved to treat multiple sclerosis (Rice et al., 2001; Heim, 2013; Ezzikouri et al., 2020).

IFNs have been explored clinically to treat different virus infections including EBOV (Rhein et al., 2015; Konde et al., 2017; Fanunza et al., 2018; Lee et al., 2019), DENV (Pires de Mello et al., 2018a), ZIKA (Ngono and Shresta, 2018; Caine et al., 2019), MERS-CoV, SARS-CoV, and SARS-CoV-2 (Cinatl et al., 2003; Tan et al., 2004; Kindler et al., 2016; Haji Abdolvahab et al., 2021). Α clinical study suggests that IFN-β-1a facilitates EBOV viral clearance and enhances survival rate (Konde et al., 2017), consistent with cell culture data that EBOV is sensitive to IFN-α or β (McCarthy et al., 2016). AG129 mice that are deficient in interferon a/β/γ signaling are more susceptible to all four serotypes of DENV (Sarathy et al., 2015; Milligan et al., 2017), suggesting the importance of IFN for DENV control. Similarly, IFN-α/β receptor (IFNAR)-deficient mice are highly susceptible to ZIKV infection (Lazear et al., 2016), and IFN-α considerately inhibits ZIKV infection *in vitro* alone or in combination with favipiravir (Pires de Mello et al., 2018b). Moreover, IFN-λ protects the female reproductive tract against ZIKV infection in mice (Caine et al., 2019).

IFNs enable to inhibit SARS-CoV or MERS-CoV in cell cultures (Cinatl et al., 2003; Falzarano et al., 2013a), and show potency in macaques infected with SARS-CoV or MERS-CoV (Haagmans et al., 2004; Falzarano et al., 2013b). Α retrospective cohort study in MERS-CoV patients shows that IFN-α-2a plus RBV results in a significant survival rate at 14 days than control supportive care (Omrani et al., 2014). However, the same treatment regimen did not benefit MERS patients in another clinical study (Al-Tawfiq et al., 2014). A preliminary, uncontrolled study shows that IFN plus corticosteroids are associated with better disease outcomes in SARS patients compared to corticosteroid treatment alone (Loutfy et al., 2003). Interestingly, a dual role of IFN for SARS pathology in mice was recently reported, while delayed IFN response correlates with severe lung immunopathology and reduced survival rate, early IFN administration ameliorates immunopathology (Channappanavar et al., 2016). IFN-α or IFN-β efficiently inhibits SARS-CoV-2 infection *in vitro* (EC50 1.35 and 0.76 IU/ml, respectively) (Mantlo et al., 2020). However, the Solidarity clinical trials launched by WHO concluded that IFN does not affect overall mortality in hospitalized COVID-19 patients. In spite of that, multiple clinical studies are still in progress to evaluate the efficacy of IFNA for COVID-19 including four phase III (NCT04492475; NCT04320238; NCT04324463; NCT04315948) and five phase IV (NCT04350671; NCT04254874; NCT04350684; NCT04291729; NCT02735707) trials.

#### Nitazoxanide

Nitazoxanide is licensed in the United States to treat parasite infection-induced diarrhea (Ortiz et al., 2001) due to the interference with the pyruvate: ferredoxin oxidoreductase (PFOR) enzyme-dependent electron transfer reaction which is essential to anaerobic energy metabolism. Nitazoxanide reduces IAV-induced duration of clinical symptoms and viral shedding in a double-blind, randomized, and placebo-controlled phase IIb/III clinical trial (Haffizulla et al., 2014). A phase III clinical trial (NCT01610245) is then initiated. The mechanism of action of nitazoxanide against IAV infection involves the inhibition in the maturation of viral hemagglutinin (HA) at the post-translational stage, thus impairing HA intracellular trafficking and insertion into the host plasma membrane (Rossignol et al., 2009). Besides, nitazoxanide is found to clinically effective to treat infections of adenovirus (Esquer Garrigos et al., 2018), rotavirus (Rossignol and El-Gohary, 2006; Teran et al., 2009), HBV (Rossignol and Keeffe, 2008), and HCV (Rossignol et al., 2008; Elazar et al., 2009; Rossignol et al., 2010). In addition, nitazoxanide enables to inhibition of other viral infections *in vitro* including HIV (Tan et al., 2012), JEV (Shi et al., 2014), ZIKV (Cao et al., 2017; Li et al., 2017c), feline calicivirus (Fumian et al., 2018), rubella virus (Perelygina et al., 2017), CHIKV(Wang et al., 2016c), paramyxovirus SeV and RSV(Piacentini et al., 2018), coronavirus MHV, MERS-CoV, and SARS-CoV-2 (Cao et al., 2015; Rossignol, 2016; Wang et al., 2020b).

Different antiviral mechanisms are involved for nitazoxanide in the context of different virus infections. Nitazoxanide prohibits SeV or RSV fusion step after entry into cells (Piacentini et al., 2018), HBV DNA replication and viral protein synthesis (Korba et al., 2008), viral RNA replication or protein processing of HCV, ZIKV, or MHV (Rossignol and Keeffe, 2008; Cao et al., 2015; Li et al., 2017c), viral morphogenesis of IAV or rotavirus (Rossignol et al., 2009; La Frazia et al., 2013). Nitazoxanide also triggers innate immune genes, like IRF1, RIG-I, or PKR, to combat norovirus or EBOV replication (Dang et al., 2018; Jasenosky et al., 2019).

HBV or HCV is susceptible to nitazoxanide treatment. An open-label small-scale clinical trial shows the preliminary efficacy of nitazoxanide in treating chronic hepatitis B (Rossignol and Keeffe, 2008). A further phase II clinical study (NCT03905655) is currently instigated. Clinical trials in hepatitis C patients show the improved SVR rate when treated alone or in combination with IFN and/or RBV (Rossignol et al., 2008; Elazar et al., 2009; Rossignol et al., 2010).

Nitazoxanide has potent antiviral activity against coronavirus. Nitazoxanide emerges as one of the most potent antivirals against MHV after drug repurposing screening (Cao et al., 2015), similar activity is observed for MERS-CoV (Rossignol, 2016) or SARS-CoV-2 (Wang et al., 2020b). A preliminary clinical study suggests the potential efficacy of nitazoxanide for COVID-19 treatment (Rocco et al., 2021). Currently, at least 18 clinical trials have been launched to test the antiviral efficacy in COVID-19 patients including five phase III (NCT04382846; NCT04392427; NCT04343248; NCT04359680; NCT04486313) and three phase IV (NCT04498936; NCT04406246; NCT04341493) clinical studies (Table 4).

## Challenges and Perspective

Currently, most of the approved antivirals are used to treat infections of HIV, HCV, HBV, and IAV, very few novel antivirals for recently emerging viruses including SARS-CoV-2, MERS-CoV, EBOV, ZIKV, and DENV. Drug repurposing has played a crucial role in pushing the approved or investigational therapeutics through clinical trials, because of higher success rate, less investment, and faster approval.

Drug repurposing is not risk-free, the success rate is reported around 30%. There are still a lot of hurdles before the repurposed drug is approved. Even though repurposed drugs could be exempted from phase I clinical trial, which mainly focuses on the drug safety evaluation, drug safety still represents one of the biggest concerns for repurposing. For instance, the safety of the drug that has been evaluated in a group of participants for the original indication does not necessarily guarantee safety in another group of people. In this scenario, drug safety may need to re-evaluate. Moreover, the dosing regimen of the repurposed drug validated previously may be different in new indications. A major obstacle to successful repurposing attributes to the higher effective concentrations in the new indication than those in the original indications. It suggests that greater harm and less benefit may be instigated. To overcome the obstacle, cocktail-based combinatorial regimens that contains at least two repurposed drugs targeting different steps of the viral lifecycle would be beneficial. In addition, drug-drug interactions may be another challenge when a repurposed drug has to use in combination with other drugs and no drug-drug interaction data is available. Thus, drug safety issue needs to be carefully appraised and addressed if necessary.

Apart from the safety issue, the intellectual property barrier is another important issue that needs to address. Commonly, drug repurposing focuses on drugs for which the patents on the original indication have been expired. For the off-patent generic drugs, a new method-of-use patent can be obtained for the new indication. However, enforceability or market exclusivity can be a major issue, as other company-manufactured generic drugs may be prescribed as off-label use for the new indication, which would be hardly prohibited. That may reduce the profit and financial incentive for drug repurposing. The off-label use can be limited if a new formulation or dosing regimen is required for the novel indication so that it cannot be easily fulfilled with the available generic versions of the drug. On the other hand, with the appropriate licensing, repurposing of drugs that are still covered by existing patent property is also achievable, even though many repurposed uses of patent drugs have been reported in the literature, which may limit the ability to gain the new patent protection. The reliable and novel evidence to support the new indication of the repurposed drug is a necessity to obtain the granted patents. Other challenges include self-medication, misuse, drug shortage, and price hike of the drugs with potential repurposed indications (Mallhi et al., 2020a; Mallhi et al., 2020b). The misuse of repurposed drugs would be devastating and should be discouraged particularly during a pandemic. The availability and affordability of these repurposed agents should also be vigilantly monitored.

With more approaches to address the safety, efficacy, and patent issues by deploying recombination therapies and reinforcing collaboration and negotiation, drug repurposing for a novel, efficient, and broad-spectrum antiviral development would strengthen the efforts to combat the emerging and re-emerging viruses.

## Glossary

BOC: bovine coronavirus
CCHFV: Crimean-Congo hemorrhagic fever virus
CHIKV: chikungunya virus
CVA16: Coxsackievirus A16
CVB1: Coxsackievirus B1
DENV: dengue virus
EBOV: Ebola virus
EBV: Epstein-Barr virus
EV71: enterovirus 71
FIPV: feline infectious peritonitis virus
HBV: hepatitis B virus
HCoV-229E: human coronavirus 229E
HCV: hepatitis C virus
HEV: hepatitis E virus
HIV: human immunodeficiency virus
hPIV: human parainfluenza virus
HRV: human rhinovirus
HSV: herpes simplex virus
HuNoV: human Norovirus
IAV: influenza A virus
JEV: Japanese encephalitis virus
JUNV: Junin virus
KFDV: Kyasanur Forest disease virus
LASV: Lassa virus
LCMV: lymphocytic choriomeningitis virus
MARV: Marburg virus
MERS-CoV: Middle East respiratory syndrome
MHV: murine hepatitis virus
MuV: Mumps virus
MV: Measles virus
OHFV: Omsk Hemorrhagic Fever virus
PRV: pseudorabies virus
RRV: Ross River virus
RSV: respiratory syncytial virus
RVFV: Rift Valley fever virus
SARS-CoV: severe acute respiratory syndrome coronavirus
SARS-CoV-2: severe acute respiratory syndrome coronavirus-2
SFTSV: Severe fever with thrombocytopenia syndrome virus
SFV: Semliki Forest virus
SINV: sindbis virus
SUDV: Sudan virus
TBEV: Tick-borne encephalitis (TBEV)
TGEV: transmissible gastroenteritis virus
VZV: varicella zoster virus
WNV: West Nile virus
YFV: yellow fever virus
ZIKV: Zika virus
